# Tuning symmetry breaking charge separation in perylene bichromophores by conformational control†

**DOI:** 10.1039/c9sc03913a

**Published:** 2019-09-30

**Authors:** Alexander Aster, Giuseppe Licari, Francesco Zinna, Elodie Brun, Tatu Kumpulainen, Emad Tajkhorshid, Jérôme Lacour, Eric Vauthey

**Affiliations:** Department of Physical Chemistry, University of Geneva CH-1211 Geneva Switzerland eric.vauthey@unige.ch; Department of Organic Chemistry, University of Geneva CH-1211 Geneva Switzerland; NIH Center for Macromolecular Modeling and Bioinformatics, Beckman Institute for Advanced Science and Technology, University of Illinois at Urbana-Champaign Urbana Illinois USA; Department of Biochemistry, Center for Biophysics and Quantitative Biology Urbana Illinois USA

## Abstract

Understanding structure–property relationships in multichromophoric molecular architectures is a crucial step in establishing new design principles in organic electronics as well as to fully understand how nature exploits solar energy. Here, we study the excited state dynamics of three bichromophores consisting of two perylene chromophores linked to three different crown-ether backbones, using stationary and ultrafast electronic spectroscopy combined with molecular dynamics simulations. The conformational space available to the bichromophores depends on the structure and geometry of the crown-ether and can be significantly changed upon cation binding, strongly affecting the excited-state dynamics. We show that, depending on the conformational restrictions and the local environment, the nature of the excited state varies greatly, going from an excimer to a symmetry-broken charge separated state. These results can be rationalised in terms of a structure–property relationship that includes the effect of the local environment.

## Abstract 2

Varying the structure of perylene-based dimers allows tuning the excited state from an excimer to a charge-separated state.

## Graphical abstract

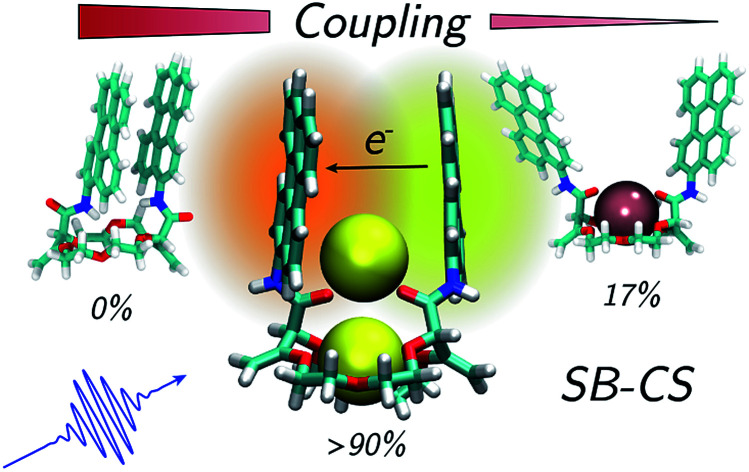

## 1 Introduction

Multichromophoric systems play important roles in biology as well as in technological applications, because they can efficiently absorb light, funnel the energy toward a defined location and separate electric charges.^1–16^ Specific optimisation of a material for one or several of these processes can be achieved by tuning the properties and the spatial arrangement of the chromophoric sub-units.^17–26^

The simplest multichromophoric array, comprising two identical sub-units, can be used to model the impact of geometry on material properties. These so-called bichromophores are particularly useful in investigating the mixing of the isoenergetic diabatic electronic states of the monomers as they approach each other.^27–33^

Since the Kasha/Davydov aggregate model in the 1960s introducing the Frenkel excitonic states for molecular dimers,^29,31^ substantial theoretical effort has been invested to understand the different states of such dimers and the effect of mutual orientation and distance.^27,28,30^ In the weak coupling limit, the dimer excited states can be theoretically described in terms of symmetry-broken local-excited (LE) states and charge-separated (CS) states. In the strong coupling limit, electronic excitation is distributed over the dimer, and the states can be described as coherent superpositions of the LE states, so-called Frenkel excitonic states, and of the CS states, resulting in charge-resonance states. In turn, Frenkel excitonic states and charge resonance states can mix to yield the experimentally observable states (Fig. S2†).^30,34^ In vacuum, these states are symmetric, but in a disordered environment, *e.g.* a liquid or a protein, local asymmetry can lead to an uneven distribution of the electronic density on the two sub-units. Therefore, a symmetric dimer with strong charge resonance character transforms into a symmetry-broken charge-transfer (CT) state with a substantial dipole moment. Depending on the local field asymmetry and on the molecular orbital overlap of the two sub-units, the CT character can be close to 100%, corresponding to a CS state. Photoinduced symmetry-breaking (SB) charge separation (CS)‡‡CS will be indistinctly used as an abbreviation for ‘charge separation’ and ‘charge separated’. could be particularly advantageous in materials for applications involving light-harvesting and charge transfer, because, first, the material can be based on a single type of chromophore and, second, the energy loss upon population of the CS state is minimal.^35–44^ This process has been suggested to be triggered by the thermal fluctuations of the solvent field, which lift transiently the degeneracy of the two diabatic CS states. As solvent relaxation takes place, the energy splitting between these two states increases, and the system is trapped in the lowest states.^42,43,45,46^

In addition to the environment, the mutual orientation and interchromophoric distance, which influence the coupling, can be expected to have a significant impact on SB. Unambiguous evidence of SB-CS between two identical molecules was obtained using a bichromophore comprising two perylene (**Pe**) moieties connected through a propyl linker (**Pe–Pr–Pe**).^42^ Unfortunately, because of the flexible linker, the **Pe** sub-units could adopt a wide range of mutual orientations and distances, hindering a detailed analysis of the structure–property relationship. More recently, several groups investigated π-stacked bichromophores, either covalently linked with stiff backbones or designed for self-assembly, and allowing for precise control of mutual geometry.^47–51^ These studies were mostly limited to closely π-stacked conformations and the differences in the dynamics and the nature of the excited states between the various structures were rather subtle.

**Fig. 1:**
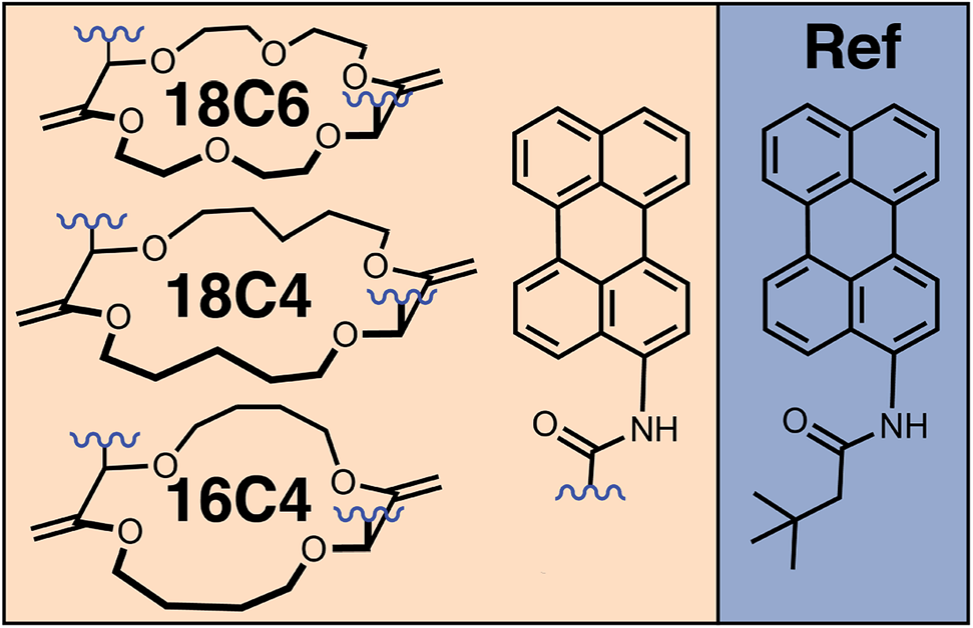
Chemical structures of the perylene (**Pe**)-based crown-ether bichromophores and of the monomeric reference (**Ref**).

Here, we combine the two approaches, first, to have control over the conformation and, second, to cover a wide range of distances and coupling energies. For this, we investigate a series of bichromophores consisting of two identical **Pe** heads connected through different crown-ether backbones (Fig. 1).^52^ Additionally to the various backbones, which already lead to distinct **Pe–Pe** distances and orientations, binding of a cation drastically changes the conformation of the macrocycle,^52–56^ and, thus, the orientational restrictions on the **Pe** heads. Spectroscopic studies are complemented with molecular dynamics (MD) simulations to estimate the most probable geometries adopted by the bichromophores at room temperature.

We begin with **18C6** and its Ba^2+^ complex in acetonitrile, first discussing the stationary electronic spectroscopy and MD simulations results to evaluate the conformational space accessible for this dimer. Based on this, we discuss the excited-state dynamics investigated using a combination of time-resolved techniques. These results are then compared with those obtained with **18C6**, **18C4**, and **16C4** and well as their Na^+^ complexes in dichloromethane to draw a comprehensive picture of the impact of conformation and local environment. We will show that the nature of the relaxed excited state is highly sensitive to these parameters, which can be used to tune the properties towards a high SB-CS yield.

## 2 Experimental part

The syntheses of **18C6**, **18C4**, **16C4** and **Ref** were previously described elsewhere.^52^ All details on the MD simulations as well as the time-resolved and stationary experiments can be found in the ESI.† Femtosecond electronic transient absorption (TA) in the visible (VIS) and 800–1600 nm (NIR) regions and broadband fluorescence up-conversion spectroscopy (FLUPS) measurements were done upon 400 nm excitation, whereas the nanosecond TA-VIS experiments were performed upon 355 nm excitation.

## 3 Results and discussion

### 3.1 **18C6** and **18C6⊂Ba2+**

#### 3.1.1 Stationary spectra and MD simulations

**Fig. 2:**
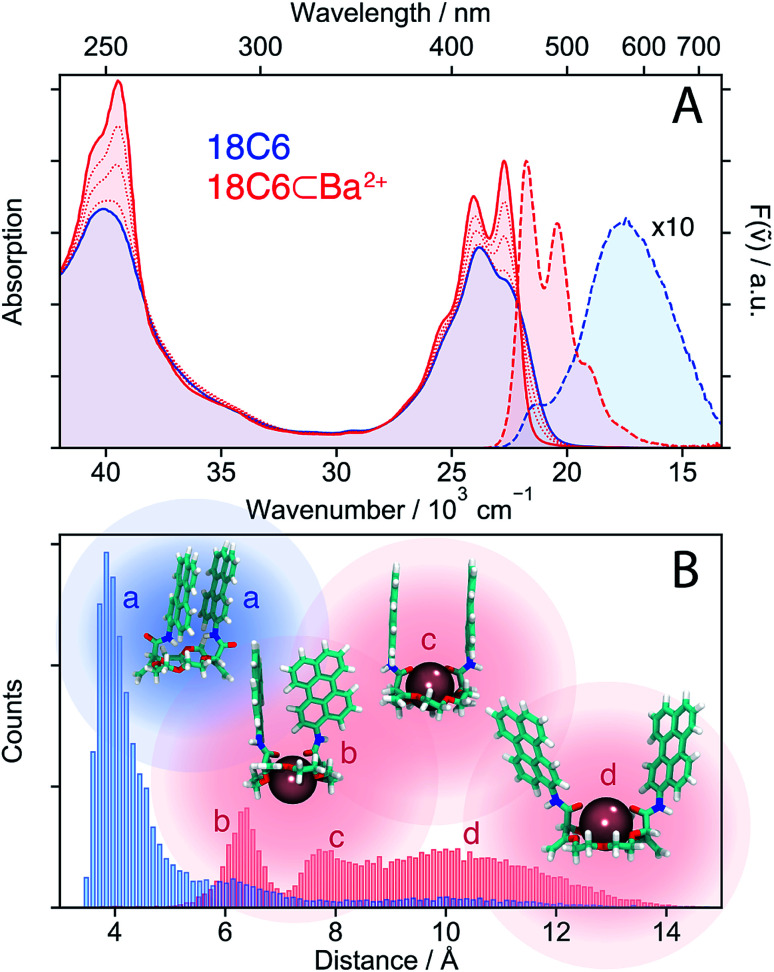
(A) Stationary electronic absorption and emission spectra of **18C6** in acetonitrile (blue) and upon addition of Ba(ClO_4_)_2_ (red). (B) Histogram of centre-to-centre distances illustrating the conformational changes upon cation binding along with representative snapshots from the MD simulations.

The electronic absorption and emission spectra of **18C6** depicted in Fig. 2A point to a substantial coupling between the **Pe** chromophores. The intensity ratio of the 0–0 and first vibronic absorption bands (*I*_0–0_/*I*_1–0_) is inverted compared to the **Ref** monomer (Fig. S5†). This is a typical signature of a strongly coupled dimer with H-type conformation.^34^ Moreover, the emission band is broad, structureless, exhibits a strong Stokes shift and resembles the fluorescence reported for the **Pe** excimer.^57,58^ Upon addition of Ba(ClO_4_)_2_, the relative intensity of the 0–0 transition increases and the vibronic structure becomes more pronounced, whereas the emission spectrum transforms into an intense band with mirror-image symmetry. These changes can be analysed assuming the binding of a single Ba^2+^ guest to the macrocycle host (**18C6**), giving **18C6⊂Ba2+** with an association constant of *K* = 4 × 10^7^ M^−1^ (Table S1, ESI†).

The similarity of the spectra of **18C6⊂Ba2+** with those of **Ref** points to a weak coupling between the two chromophores and suggests that absorption and emission involve LE states. In the absence of Ba^2+^, emission occurs from a lower-energy state and has smaller oscillator strength than the LE emission, as expected for an excimer state.

MD simulations were performed to obtain a quantitative picture of the conformational space accessible to **18C6** and **18C6⊂Ba2+**. Geometrical parameters, namely the **Pe–Pe** centre-to-centre distance, the angle between the long axes of the **Pe** heads, the angle between the **Pe** planes and the slip angle, were extracted from the ensuing trajectories (see Fig. S1† for the definition). As depicted in Fig. 2B, the two **Pe** sub-units in **18C6** are closely π-stacked, in agreement with the significant coupling inferred from the stationary spectra. The angle between the long axes of the **Pe** heads is distributed around 30° (Fig. S9†), in line with previous quantum-chemical calculations predicting a ground-state minimum geometry of the **Pe** dimer with *D*_2_ symmetry (Fig. S3†).^30^ This indicates that, in contrast to a stiff backbone, the crown-ether macrocycle allows the **Pe** chromophores to adopt the most stable dimer configuration.

The effect of Ba^2+^ on the spectra can be explained by conformational changes of the **18C6** host upon cation binding,^53,59^ as illustrated by representative snapshots in Fig. 2B. Along with the oxygen atoms of the crown-ether, the two amide oxygens are involved in metal binding and cause a rotation of the **Pe** sub-units leading to an increase of the **Pe–Pe** distance to >6 Å. These simulations point to a broad distribution of geometries, especially in the presence of Ba^2+^, with distinct sub-populations, featuring T-shape and H-type conformers (snapshots b and c in Fig. 2B, respectively).

In contrast, the two-dimensional excitation/emission maps (Fig. S6 and S7†) suggest a narrowing of the distribution upon binding of a cation. This apparent contradiction with the MD simulations can be accounted for by the substantial increase of **Pe–Pe** distance upon complexation. Indeed, small structural changes at short distances, where the molecular orbitals of both sub-units overlap, have a stronger impact on the spectrum than large changes at long distances. This short-range sensitivity stems from CT-mediated exciton coupling (*J*_CT_), which, in addition to the Coulombic interaction (*J*_C_), predominates at close distances as it depends on the overlap of the frontier MOs of the two **Pe** sub-units.^34^ As shown in Fig. S9,† the flexible crown-ether backbone allows for a wide range of angles between the **Pe** long axes, altering *J*_CT_. On the other hand, if the **Pe–Pe** distance is too large for significant orbital overlap, the spectra are much less influenced by subtle conformational changes, explaining the excitation wavelength independent emission spectra of **18C6⊂Ba2+**.

#### 3.1.2 Time-resolved spectroscopy

##### 18C6

**Fig. 3:**
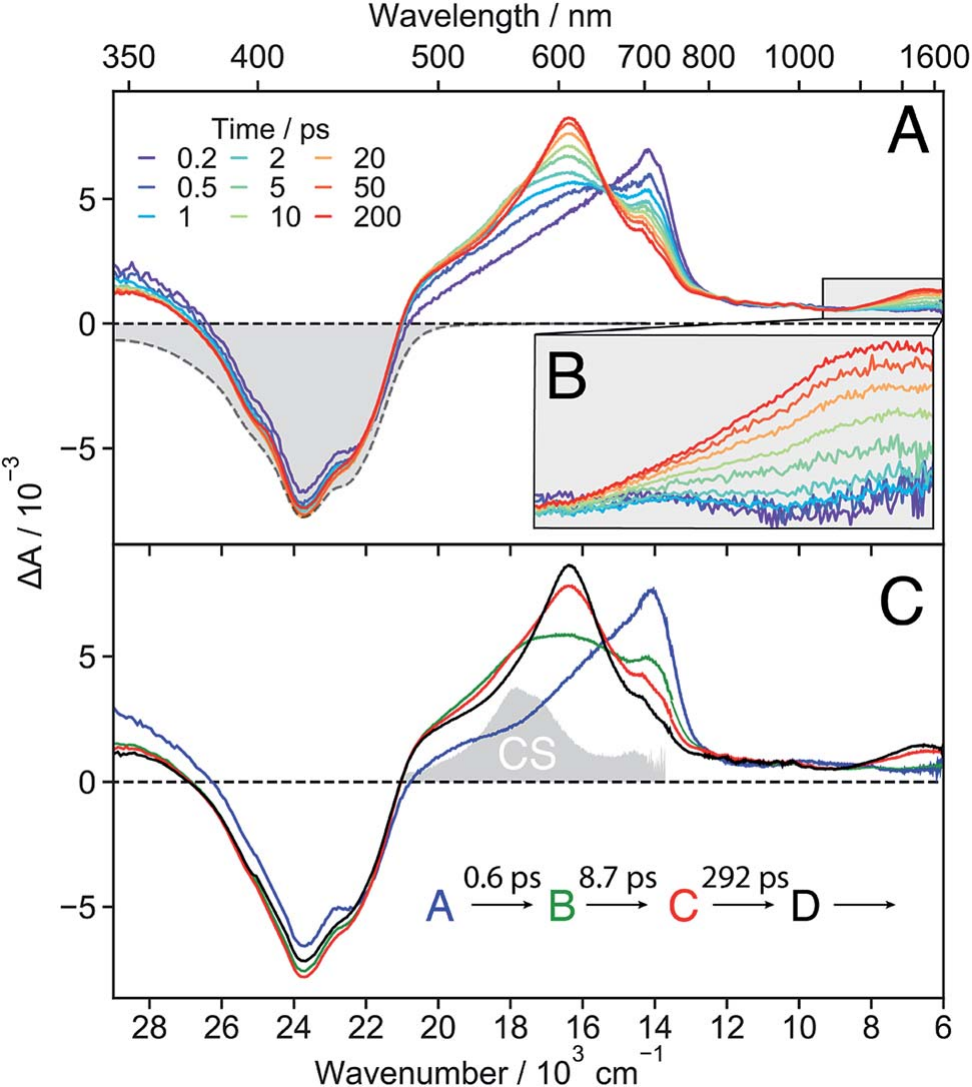
(A) Transient absorption spectra measured at different time delays after excitation of **18C6** in acetonitrile together with the negative stationary absorption spectrum (grey). (B) Zoom in on the NIR region. (C) Evolution associated difference spectra (EADS) together with the corresponding time constants. The absorption spectrum of the charge-separated state measured with **18C6⊂Ba2+** (*vide infra*) is depicted in grey to illustrate the spectral resemblance to the shoulder in EADS B and C.

The early TA spectra measured with **18C6** in acetonitrile are dominated by a positive band peaking at around 700 nm and a negative band centred at about 420 nm (Fig. 3A). The positive band resembles the S_*n*_ ← S_1_ absorption band of **Pe**,^60,61^ but is significantly broader on the blue side. The negative band coincides with the stationary absorption spectrum and can be assigned to the ground-state bleach (GSB). No contribution from stimulated emission (SE) is observed. Within a few ps, the 700 nm band transforms into a band centred around 600 nm and, in parallel, a weak positive band rises around 1530 nm (Fig. 3B). Both bands decay concomitantly with the GSB on the tens of ns timescale (Fig. S16†).

These data were analysed globally assuming a series of four exponential steps and the resulting evolution associated difference spectra (EADS) and time constants are illustrated in Fig. 3C. These EADS and time constants cannot be systematically assigned to well-defined species/states and kinetic processes, respectively. Nevertheless, they give a semi-quantitative picture of the spectral dynamics and of the timescales on which they occur.^62^

This analysis reveals that the transformation of the 700 nm band (EADS A) involves an intermediate stage, accounted for by EADS B and C, which are characterised by a shoulder at 560 nm and a weaker intensity than the long-lived 600 nm band (EADS D). The latter is very similar to the TA spectrum observed with highly concentrated **Pe** solutions in toluene and attributed to the excimer.^63^ Previous quantum-chemical calculations of the cofacially stacked **Pe** dimer predict that the equilibrium geometry of the lowest excitonic state, namely the excimer,^64^ adopts a *D*_2h_ geometry (Fig. S3†). During structural relaxation towards the excimer, the lowest energy absorption band shifts from the mid- to the near-IR region.^30,65^ Based on this, the 1530 nm band can also be assigned to an intramolecular excimer. In the following, the term excimer will be strictly used to designate the relaxed S_1_ excited state of the dimer. The 600 and 1530 nm excimer bands increase in intensity as the mutual orientation of the **Pe** heads evolves from the *D*_2_ to the more stable *D*_2h_ geometry. The MD simulations indicate that the macrocycle allows for the eclipsed *D*_2h_ conformation, although it is not the most stable ground-state structure. Similar NIR excimer bands have been reported for perylene and perylenediimide dimers.^51,66^

The 560 nm shoulder in EADS B and C is not visible in the medium and low polarity solvents, dichloromethane (DCM) and toluene (Fig. S17†). Moreover, it coincides with the absorption band of the CS state (*vide infra*) with the **Pe** radical cation and anion absorption bands at 540 and 580 nm, respectively (grey in Fig. 3C).^42,61,67^ As this feature is not very pronounced and the coupling between the two **Pe** moieties, hence their molecular orbital overlap, is large, we attribute it to a SB-CT state rather than to a SB-CS state. Hereafter, we will call CS state, a state with clear spectral signature of ions, although this does not always imply a full CT character.

Given the distribution of conformations, a fraction of the excited-state population might directly form the excimer without going through the CT state. This appears to be the dominant pathway in the less polar solvents. Such distribution of geometries can also explain why features of both the CT and the excimer states are visible in EADS C.

**Fig. 4:**
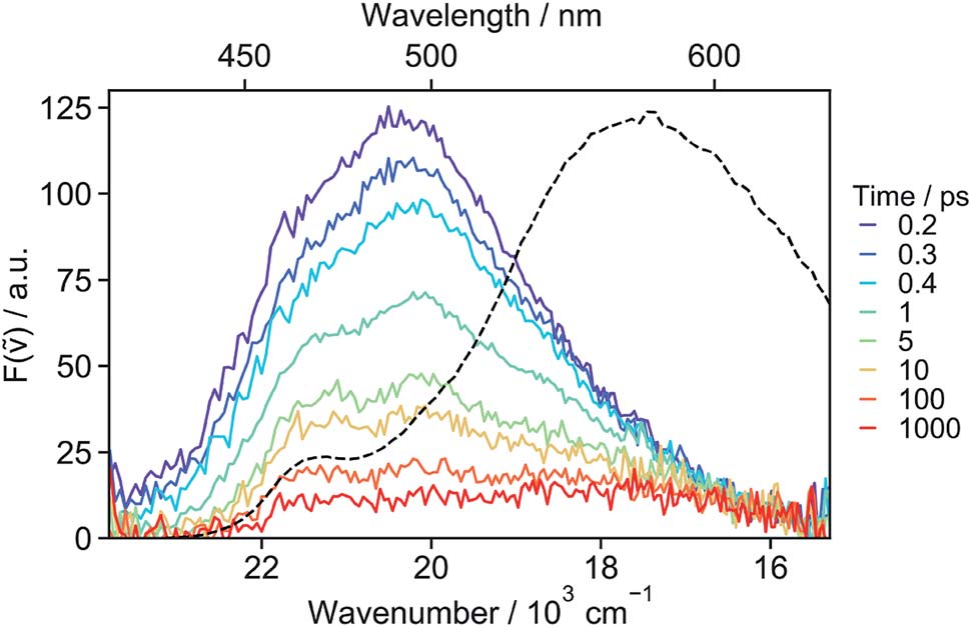
Fluorescence spectra recorded at various time delays after 400 nm excitation of **18C6** in acetonitrile and stationary emission spectrum (dotted line).

The absence of SE in the early TA spectra suggests that these spectra (or EADS A) are not those of the initially photo-populated state, because excitation is done at a transition with a large dipole moment. This points to an ultrafast conversion from the initially populated bright state to a quasi-dark state, which equilibrates to the weakly emissive excimer. To unravel the processes occurring prior to the excimer formation, we applied time-resolved fluorescence spectroscopy, which has the advantage of emphasizing the excited states with a large emission dipole moment. As shown in Fig. 4, the time-resolved emission spectra differ significantly from the stationary spectrum. The early fluorescence band peaks at around 490 nm and its area is smaller by about one order of magnitude compared to that of the LE emission recorded under the same experimental conditions (*vide infra*), indicative of a significantly smaller transition dipole moment. This is fully consistent with the absence of a SE band in the TA spectra (Fig. 3). The 490 nm band transforms on similar timescales as the A → B → C steps in the TA data to a hardly visible band resembling the stationary excimer spectrum. As the ground-state population does not recover during this time interval, the decay of the fluorescence intensity can be interpreted as a decrease of the emission dipole moment. In the weakly polar toluene, the fluorescence decay is comparatively slower than in ACN (Fig. S24†). This can be explained by the fact that SB-CT accelerates the decrease of the oscillator strength in ACN, but does not prevent the formation of the excimer. SB occurs on the timescale of solvent fluctuations, whereas structural relaxation is comparatively slower.^68–71^

**Fig. 5:**
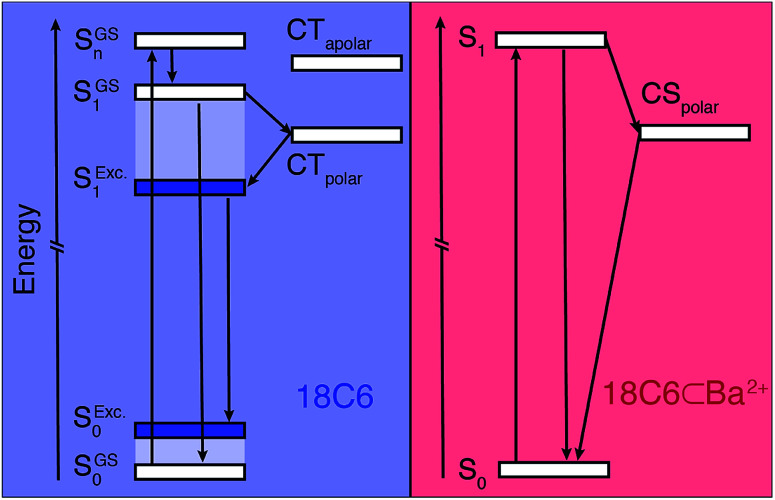
Energy level diagrams illustrating the excited-state dynamics of **18C6** (left) and **18C6⊂Ba2+** (right). The superscripts ‘GS’ and ‘Exc.’ stand for the ground-state equilibrium geometry and excited state equilibrium geometry (excimer), respectively. The S states of **18C6** (left) are delocalised whereas that of the **18C6⊂Ba2+** is mostly localised.

The ensemble of experimental results suggests that photoexcitation of **18C6** leads to the population of a higher excitonic state with a large oscillator strength (Fig. 5, left). It is followed by an internal conversion to the lowest excitonic state, S_1_, that is too fast to be resolved experimentally (*τ* < 100 fs). This ultrafast internal conversion prevents major geometrical reorganisation prior to population of the S_1_ state, whose initial geometry is thus similar to that in the ground state (S^GS^_1_), *i.e.* mainly with a *D*_2_ mutual orientation of the **Pe** sub-units. Fluctuations of the surrounding polar solvent leads to SB and confer a significant amount of CT character. However, as structural relaxation takes place, this state relaxes to the excimer equilibrium geometry, characterised by a lower CT character.

In toluene, SB-CT is not operative and the S_1_ state relaxes directly to the excimer. The excimer ultimately decays on a ns timescale (Fig. S16†), which is consistent with the large energy gap relative to the ground state and the vanishingly small emission transition dipole moment of a H-type dimer.

##### 18C6⊂Ba2+

**Fig. 6:**
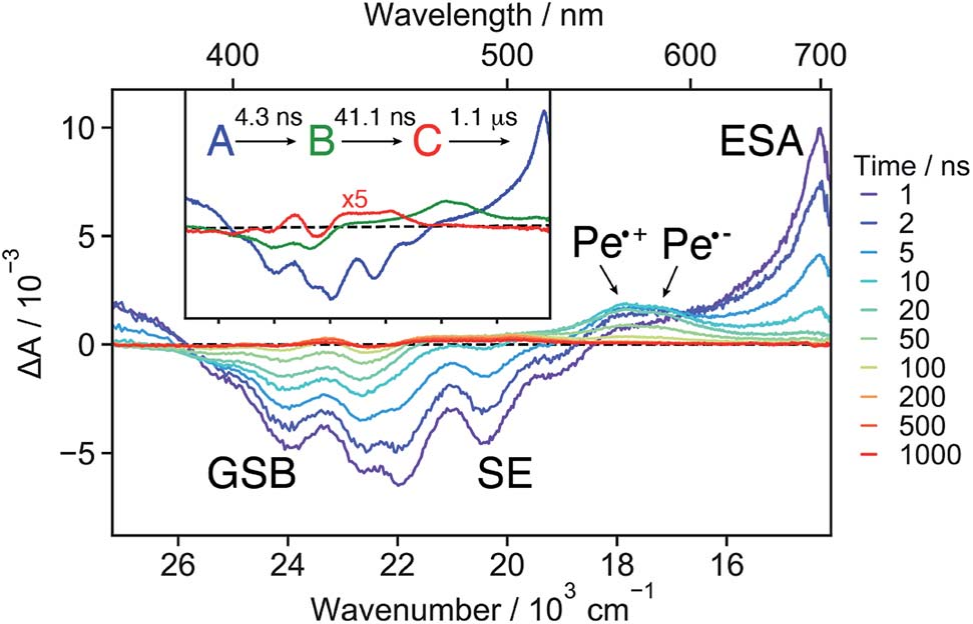
Transient absorption spectra measured with **18C6⊂Ba2+** in acetonitrile at different time delays after 355 nm excitation. The evolution associated difference spectra (EADS) obtained from a global analysis assuming a sequential model are shown in the inset.

The TA spectra recorded at early time with **18C6⊂Ba2+** exhibit a positive band around 700 nm and a negative band due to both GSB and SE (Fig. 6 and S15†). The spectra resemble those measured with **Ref** in DCM (Fig. S19†) as well as those reported for **Pe**.^60,72^ This implies that excitation is mostly localised on a single **Pe** sub-unit. Apart from small shifts of the SE and ESA bands attributed to vibrational relaxation, (Fig. S15†)^73–75^ no significant dynamics occur during the first ns after excitation, in agreement with the weak coupling suggested by the stationary spectra and the MD simulations.

Both the 700 nm and SE bands decay on a few ns timescale in parallel with the rise of a band peaking at around 550 nm with a shoulder at 580 nm. This band resembles the sum of the **Pe** radical cation and anion bands.^42,61,67^ It is much more pronounced than the shoulder observed with **18C6** in ACN (Fig. 3). Consequently, it is attributed to a SB-CS state, in agreement with the smaller coupling of the chromophores. It evolves in a few tens of ns to a very weak residual spectrum, that in turn decays on the μs timescale. The last spectrum is similar to that of the triplet LE state of **Pe**.^72,76^

These data were analysed globally assuming a series of exponential steps and the resulting EADS and time constants are illustrated in the inset of Fig. 6. The A → B step can be attributed to the LE → CS process. A CS time constant of 25 ns is estimated from the A → B time constant and the fluorescence lifetime of **Ref**. This value, which should be considered as an average over a distribution of different conformers, points to a 17% CS yield. Finally, the SB-CS state decays by charge recombination predominantly to the neutral ground state in about 40 ns.

SB-CS in **18C6⊂Ba2+** is significantly slower than in the above-mentioned flexible **Pe–Pr–Pe**, with 12 and 130 ps time constants.^42^ The conformational space accessible to the **18C6⊂Ba2+** is shifted towards larger distances compared to **Pe–Pr–Pe** in which the **Pe** sub-units can approach to less than 4 Å. The increased distance results in weaker electronic coupling and, thus, slower CS. Most probably, structural fluctuations are required for the subpopulations with the largest **Pe–Pe** distance for CS to be operative within the lifetime of the LE state.

The time-resolved emission spectra recorded with **18C6⊂Ba2+** resemble the stationary spectrum (Fig. S23†). During the first ns after excitation, these spectra show relatively little dynamics, apart from some early band narrowing, due to vibrational relaxation already observed in the SE.^74,75^ The area is larger by approximately one order of magnitude compared to those measured with **18C6** (Fig. 4). This points to an emission from a LE state with a large transition dipole moment, in agreement with the presence of a SE band in the TA spectra.

The excited-state dynamics of **18C6⊂Ba2+** are thus similar to those expected for two weakly coupled **Pe** chromophores in a polar environment. Initial population of a LE state is followed by SB-CS, in addition to the radiative and non-radiative relaxation to the ground state (Fig. 5 right).

### 3.2 Searching for the “sweet spot”

As shown above, the conformational restrictions imposed by the binding of Ba^2+^ to **18C6** render the eclipsed, close-distance *D*_2h_ excimer conformation inaccessible. Therefore, the SB-CS state becomes the lowest excited state in polar solvents. However, the slow electron transfer caused by the weak coupling between the chromophores leads to a CS yield of only 17%. On the other hand, in the absence of cation, the π-stacked conformation undergoes ultrafast but incomplete SB-CS in polar solvents, and the excited state relaxes rapidly to the excimer.

Is there a “sweet spot” geometry between this two cases where fast and long-lived CS is possible? To answer this question, we investigated **18C4** and **16C4**, for which the conformational space accessible to the **Pe** chromophores differ from that of **18C6**. Instead of Ba^2+^, which does not bind to all three crown-ethers, we used a Na^+^-salt (NaBAr_F_, sodium tetrakis[3,5-bis(trifluoromethyl)phenyl]borate), which is soluble in the medium polarity solvent, DCM. For comparison, **18C6** was also studied in DCM with Na^+^.

#### 3.2.1 Stationary spectra and MD simulations

**Fig. 7:**
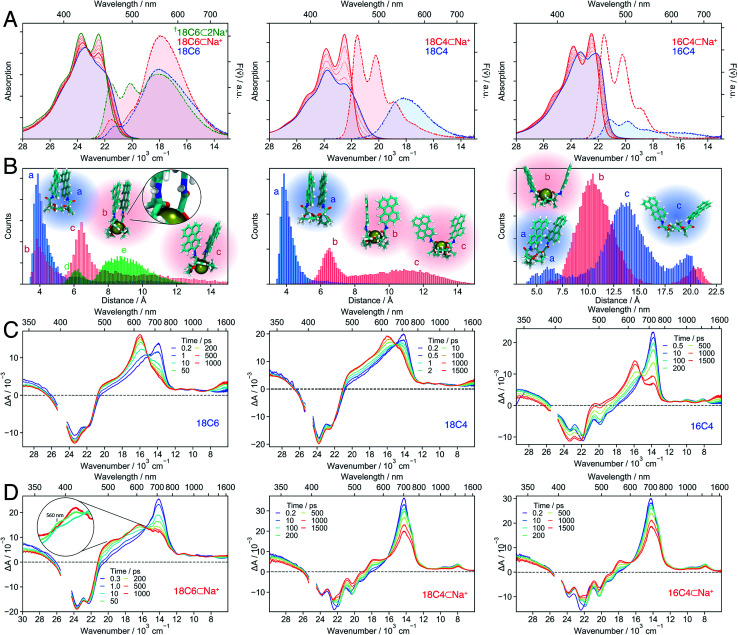
Stationary electronic spectra (A), MD simulations (B) and transient absorption spectra of the hosts (C) and host–guest complexes (D) with Na^+^ in DCM (left: **18C6**; middle: **18C4**; right: **16C4**). The emission spectra can be quantitatively compared since the same solution and conditions were used and the changes in molar absorption coefficient at the excitation wavelength (400 nm) upon cation binding do not change significantly. The † indicates that the spectra are due to a mixture of 1 : 1 and 1 : 2 complexes. The green histogram in the left panel of B corresponds to **18C6⊂2Na+** with the snapshots d and e shown in Fig. 8B.

The electronic spectra of **18C4** in DCM show indications of a strong coupling between the **Pe** moieties, similar to those observed with **18C6** (Fig. 7A). This is supported by the MD simulations, which predict the predominance of closely stacked conformations (Fig. 7B). The presence of LE-emission in **18C6** but not **18C4** can be explained by the sub-populations with **Pe–Pe** distance >6 Å, which is only significant for the former.

In contrast, the *I*_0–0_/*I*_1–0_ ratio in the absorption spectrum of the bichromophore host with the smallest crown-ether, **16C4**, is markedly larger and points to a weaker coupling relative to the other two macrocycles. Similarly, the fluorescence spectrum is dominated by the monomeric LE emission. The MD simulations suggest that **16C4** exhibits the broadest distribution of geometries and largest **Pe–Pe** distances. This is due to geometrical restrictions, which most likely hinder access towards the close-contact *D*_2_ conformation, as reflected by the angle between the long axes of the **Pe** sub-units that deviates largely from 30° (Fig. S13†). The small difference in *I*_0–0_/*I*_1–0_ ratio between **16C4** and the monomeric **Ref** most likely originates from the small subpopulation with **Pe–Pe** distance of <7.5 Å.

Upon addition of Na^+^, the vibronic structure of the absorption band of all three bichromophores becomes more pronounced. Moreover, the emission spectra of **18C4** and **16C4** are dominated by LE fluorescence. In the case of **18C6**, the broad excimer-like band remains clearly visible even at the highest Na^+^ concentration. Interestingly, the intensity of this band first increases with Na^+^ concentration and then decreases.

The spectral changes measured with **18C4** and **16C4** could be reproduced by assuming 1 : 1 association model with the association constants and spectra given in Table S1 and Fig. S4.† MD simulations indicate that, upon complexation, the amide oxygens of the **Pe** chromophores, pointing originally towards the outside, bind to the cation. This requires a rotation of the amide groups, which in turn leads to the rotation of the whole **Pe** heads and to a concomitant increase of **Pe–Pe** distance. The simulations also reveal that complexation of **16C4** results in a decrease of the average **Pe–Pe** distance. Nevertheless, distances shorter than 7.5 Å are geometrically unfavourable in **16C4⊂Na+**, and are only accessible in **16C4** alone. This supports the above hypothesis that the difference in *I*_0–0_/*I*_1–0_ ratio between **16C4** and **Ref** is only due to the small sub-population with **Pe–Pe** distance of <7.5 Å.

In contrast, a 1 : 2 association model had to be assumed to reproduce the spectral changes measured with **18C6** upon addition of Na^+^. The 1 : 1 association constant is similar to those found for the other two crown-ethers (Table S1†), whereas the 1 : 2 association constant is smaller by three orders of magnitude. The binding of a single Na^+^ to **18C6** leads to a small increase of the *I*_0–0_/*I*_1–0_ ratio as well as a ∼50% increase of the excimer-like emission band. According to the MD simulations, only one of the **Pe** heads is rotated and its amide oxygen atom binds to the cation and to the NH group of the other chromophore (snapshot b in Fig. 7B left). Such asymmetric binding can be expected to result in dissimilar properties of the two **Pe** sub-units and thus to an intrinsic SB. The magnitude of this effect was estimated by inspecting the changes in the stationary spectra of **Ref** upon addition of Na^+^. As shown in Fig. S5,† in the presence of Na^+^, both the absorption and emission bands of **Ref** shift to higher energy by about 500 cm^−1^ and the vibronic structure is more pronounced. As expected, the association constant is two orders of magnitude smaller than for the crown-ether hosts (Table S1†). The binding of Na^+^ to the amide oxygen of **Ref** rises the energy of its first singlet excited state, most probably because the electron-donating ability of the amide substituent is switched off.^77^ As a consequence, **Ref** becomes similar to **Pe** upon complexation.

**Fig. 8:**
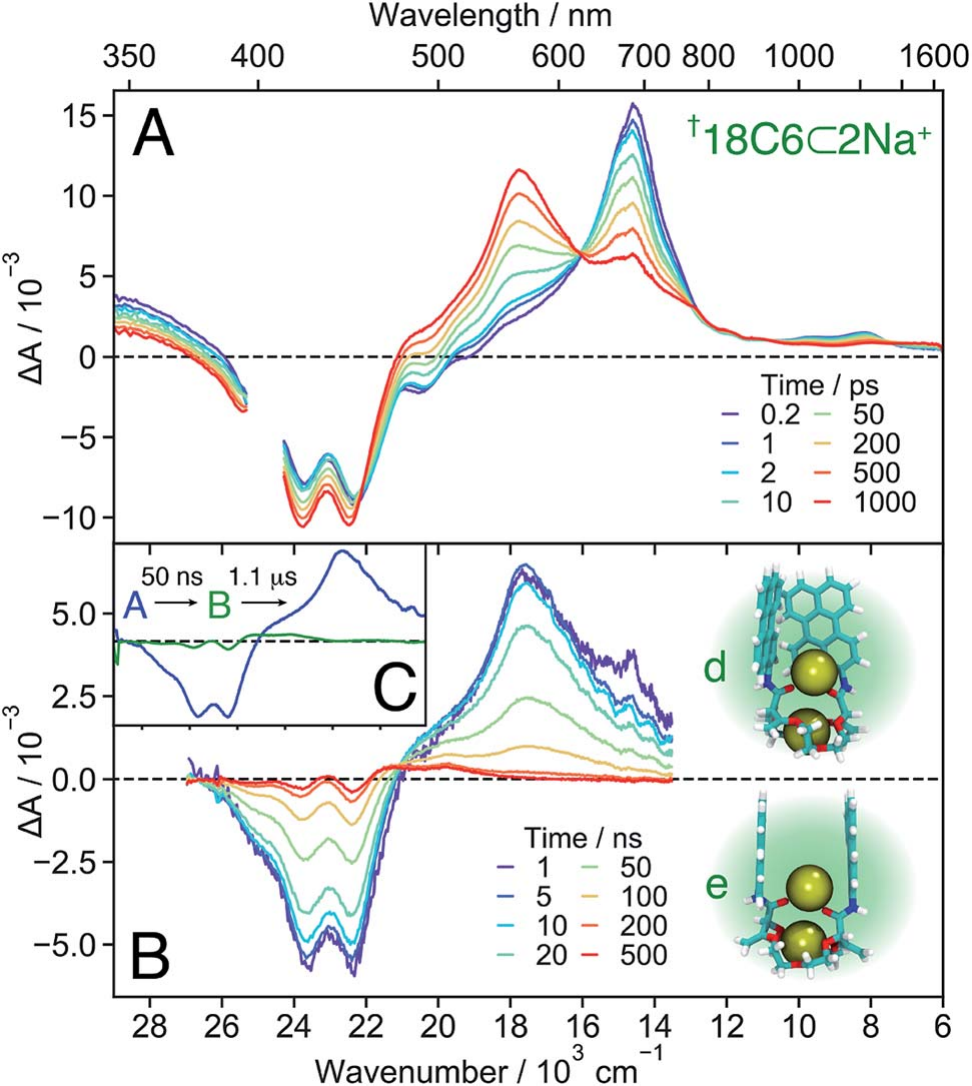
Transient absorption spectra measured with †**18C6⊂2Na+** in DCM on the femtosecond (A) and nanosecond (B) timescale, together with snapshots obtained from MD simulations (see also Fig. 7B left). (C) Evolution associated difference spectra (EADS) obtained from a global analysis of the nanosecond transient absorption data.

Even though full conversion to **18C6⊂2Na+** could not be achieved due to the limited solubility of NaBAr_F_ in DCM, the absorption spectrum of **18C6⊂2Na+** could be obtained from the global analysis and resembles those of the 1 : 1 complexes of the two other crown-ethers (Fig. S4†). The *I*_0–0_/*I*_1–0_ ratio of the absorption spectrum and the presence of the monomeric LE emission point to a decrease of the coupling between the **Pe** moieties upon complexation of a second Na^+^. A stable 1 : 2 complex with two Na^+^ was found in the MD simulations (Fig. 7B left), with two representative snapshots shown in Fig. 8A. One cation is bound to the crown-ether, whereas the other is symmetrically bound to the two amide oxygens. The second Na^+^ is possibly further stabilized by cation–π interaction.^78,79^ Even though this interaction is predicted to be smaller if the cation is not centred above the π surface, it can still have a stabilizing effect.^80^ The binding of a second cation leads to a **Pe–Pe** distance larger than 5.5 Å, in agreement with the smaller coupling inferred from the spectra.

Since the 1 : 1 and 1 : 2 association constants differ by three orders of magnitude, a symmetrically π-stacked conformer (**18C6**) and a π-stacked conformer with local asymmetry (**18C6⊂Na+**) can be quantitatively produced by adjusting the concentration of NaBAr_F_ in DCM. Further addition of Na^+^ leads to a mixture of **18C6⊂Na+** and **18C6⊂2Na+**. The excimer-like fluorescence measured at the highest Na^+^ concentration is assigned to **18C6⊂Na+** and not to **18C6⊂2Na+**, as discussed in the ESI (Fig. S8†).

#### 3.2.2 Time-resolved spectroscopy

##### Bichromophore hosts

The TA spectra measured with all three bichromophore hosts in DCM (Fig. 7C) confirm the different extent of coupling revealed by the stationary spectra and MD simulations. No SE band can be detected in the **18C6** and **18C4** spectra, whereas it is clearly visible up to 1 ns in those recorded with **16C4**. Like for **18C6** in ACN (Fig. 3), the spectra are first dominated by the 700 nm band and then show the build-up of the excimer bands around 600 nm and above 1500 nm. However, the 560 nm shoulder, attributed to the SB-CT state, is no longer visible in the less polar DCM. The rise of the excimer bands occurs within a few tens of ps for **18C6** and **18C4** but is distinctly slower for **16C4**, taking place on a 1 ns timescale. This difference is fully consistent with the MD simulations that predict much larger **Pe–Pe** distances in **16C4** than in the other two hosts. Thus, larger structural reorganisation is required to relax to the excimer geometry.

##### 1 : 1 Host–guest complexes

The TA spectra measured with both **18C4⊂Na+** and **16C4⊂Na+** in DCM exhibit a slow decay of the 700 nm and SE bands and a concomitant rise of a band around 560 nm, that is attributed to a SB-CS state (Fig. 7D). The same 560 nm band is found a few tens of ns after excitation of **Ref** in DCM (Fig. S20†). It results from the self-quenching of **Ref** by SB-CS, as already observed with **Pe**.^81,82^ The fact that the **Pe** cation and anion maxima cannot be distinguished here is probably due to the lower polarity of DCM compared to ACN. Another evidence for the assignment of this band to a SB-CS state of **18C4⊂Na+** and **16C4⊂Na+** rather than to an excimer is the absence of a NIR band.

The TA spectra measured with **18C6⊂Na+** in DCM show the very fast build up of a shoulder around 560 nm, similar to that measured with the host alone in ACN (Fig. 3). We also attribute it to a CT rather than to a CS state, as it rapidly decays concomitantly with the rise of a band around 600 nm. The latter differs significantly from the excimer band, and is not accompanied by a NIR band. This suggests that the relaxed excited state of **18C6⊂Na+** differs significantly from the relaxed excimer state observed with the **18C6** alone. This is further supported by the stationary fluorescence spectrum of **18C6⊂Na+**, which is more intense than that of **18C6**, indicative of a stronger transition dipole moment. This state is tentatively interpreted as an excimer-like state with a geometry departing from that of the eclipsed *D*_2h_ excimer. Access to the latter geometry is probably hindered by the presence of Na^+^.

Occurrence of SB-CT for **18C6⊂Na+** in DCM, despite short **Pe–Pe** distances as in the host alone, could arise from the above-discussed asymmetry caused by the binding of Na^+^ to a single amide oxygen observed in the MD simulations. Without this intrinsic asymmetry, SB-CT in the medium polar DCM would be probably too slow to compete with the structural relaxation towards the excimer. The asymmetric binding of Na^+^ and the ensuing differences in electronic structure of the two **Pe** heads could be a further reason why the relaxed excited state of the **18C6⊂Na+** complex differs spectrally from the excimer formed with the hosts alone.

##### 18C6⊂2Na+

The TA spectra recorded with **18C6** at the highest Na^+^ concentration are presented in Fig. 8A. Based on the association constants and the molar absorption coefficients, the probability of the 1 : 2 complex to be excited at 400 nm is twice as large as that of the 1 : 1 complex. These spectra reveal that the build up of the 560 nm band is not followed by a rapid transformation to the 600 nm band as observed with the 1 : 1 complex, but decays to the ground state on a 50 ns timescale. This band is, thus, ascribed to a SB-CS state. The TA spectra shown in Fig. S18† illustrates how the nature of the excited state of **18C6** and its dynamics change upon increasing Na^+^ concentration.

In contrast to all other conformers, SB-CS is nearly quantitative as indicated by the absence of ground-state recovery. The slight increase of the bleach is due to the decay of the overlapping 700 nm band and is expected for a nearly quantitative conversion.^72^ The high CS yield can be explained by a faster SB-CS in **18C6⊂2Na+** compared to the 1 : 1 complexes of the other two macrocycles. This increased rate is assigned to the larger coupling between the **Pe** sub-units, as suggested by the MD simulations (Fig. 7B). They predict two main subpopulations: one with a ∼6 Å **Pe–Pe** distance and a T-shape conformation, and one around 8–10 Å with a face-to-face conformation. The latter structure, with significant molecular orbital overlap and, thus, an enhanced CS rate, is not predicted for **18C4⊂Na+** and **16C4⊂Na+**. Indeed, the **Pe–Pe** distances in the face-to-face configurations are on average significantly larger in these two 1 : 1 complexes than in **18C6⊂2Na+**. The second Na^+^ interacts with both the amide oxygens and the π-surfaces of the two **Pe** heads and ensures a relatively close distance between the chromophores. The MD simulations indicate that, on average, the ions are located at equal distances from the two **Pe** heads but the difference in these distances fluctuates significantly (Fig. S11†). These transient departures from symmetry due to the cations probably also contribute to the fast SB-CS. The cations should also participate to the stabilisation of the SB-CS state, additionally to the solvent. This stabilisation should mainly be due to Coulombic interactions and result in the translocation of the cation(s) towards the negatively-charged **Pe** head.

Finally, the second Na^+^ also prevents further approach of the chromophores and excimer formation. Therefore, the CS state recombines relatively slowly to the ground state with a 50 ns time constant (Fig. 8B), very similar to that found for **18C6⊂Ba2+** in ACN. The nearly “sweet spot” geometry, allowing fast and long-lived SB-CT, is realised with the **18C6⊂2Na+** complex.

### 3.3 Structure–property relationship

**Fig. 9:**
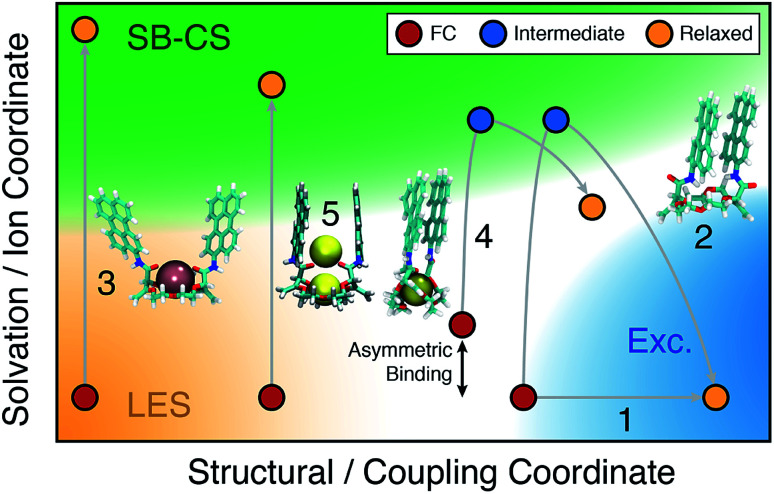
Schematic two-dimensional representation of the relaxation pathways (gray lines) on the S_1_ surface of the **18C6** host, 1 : 1 and 1 : 2 complexes (see text for details). FC: initial location at the ground-state geometry; LES: locally excited state; Exc.: excimer. The coloured area refer to the nature of the excited state.

A comprehensive picture of the impact of structure on the nature of the excited state of **Pe**-based bichromophores can be drawn from this ensemble of results. Three main factors have to be considered: (i) the coupling between the two chromophores at the ground state geometry, (ii) their conformational flexibility, and (iii) their local environment, which includes the solvent and the cation(s). Their effect on the excited-state dynamics can be visualised by splitting the reaction coordinates into a structural and a solvation coordinate as depicted in Fig. 9. The structural coordinate describes both the **Pe–Pe** distance and their mutual orientation and is directly related to the coupling, increasing from left to right.

The extent of coupling at the ground state geometry (factor (i)) determines the initial position along this coordinate, whereas the conformational flexibility (factor (ii)) affects the capacity to evolve along this coordinate. Finally, the solvation coordinate plays a crucial role in the SB-CS process. Here, it includes the solvent polarisation, which depends on the solvent polarity and also the asymmetry in cation position in the complex (factor (iii)). The extent of CT character of the excited state increases along this coordinate.

For example, the excited-state dynamics of **18C6** in toluene can be represented by a simple evolution along the structural coordinate (trajectory 1 in Fig. 9). By contrast, in ACN, the system first moves along the faster solvation coordinate to an intermediate SB-CT state before evolving along the structural coordinate towards the more stable excimer state (trajectory 2). Polar solvation does not suffice to stabilise a significant CT character in a compact dimer. Thus, solvent relaxation around the weakly polar excimer corresponds to a ‘backward motion’ along the solvent coordinate. Upon complexation with Ba^2+^, the structural coordinate is essentially frozen at a weak coupling position and the reaction can only proceed along the solvent coordinate towards the SB-CS state (trajectory 3). Replacement of Ba^2+^ by Na^+^ and ACN by DCM, leads to trajectory 4 where the relaxed state should have some excimer character as well as some asymmetry due to the presence of the cation.

Finally, in the “sweet spot” geometry, the structural coordinate should be locked in an intermediate position, where coupling is large enough to ensure fast SB-CS, but sufficiently small to prevent the collapse to an excimer-like state. This condition seems to be fulfilled for **18C6⊂2Na+** (trajectory 5). In DCM, the evolution along the solvation coordinate is limited but is compensated by the presence of the ions. If the interactions with the ions are not the same for the two **Pe** heads, the latter can no longer be considered as two identical chromophores. This leads to an intrinsic SB and favours CS in one direction with respect to the other. In the absence of ions, symmetry breaking is only driven by the thermal fluctuations of the solvent field, that transiently lifts the degeneracy of the two CS states. Consequently, the increase of the driving force arising from such asymmetric ion–chromophore interactions plays in favour of a faster SB-CS.

The results obtained with the other two bichromophores can be rationalised in a similar way. The hosts alone follow trajectories that involve mostly the structural coordinate (trajectory 1), whereas the trajectories of the 1 : 1 complexes are close to trajectory 3.

## 4 Conclusions

These crown-ether based **Pe** dimers allowed establishing a comprehensive structure/property relationship. Given their flexibility, MD simulations proved to be crucial for rationalising the spectroscopic observations. The results presented here demonstrate that these bichromophores allow the coupling to be varied in a relatively well-controlled fashion over a wide range, contrary to previous studies. This is achieved by first changing the size and nature of the macrocycle. However, as all three crown-ethers allow for a relatively large conformational flexibility, the excited bichromophores finally equilibrate to an intramolecular excimer inhibiting the population of the CS state.

The second way to modify the coupling is upon complexation with one or two cations. Cation binding not only changes the ground-state geometry and decreases the coupling, but also prevents the close approach of the **Pe** heads and, thus, excimer formation. As a consequence, the excited state relaxes to a SB-CS state. The exception is **18C6⊂Na+** where close **Pe–Pe** distances are still accessible.

Our results also suggest that symmetry breaking in the complexes is facilitated by the imbalanced interactions of the ion(s) with the two **Pe** heads. Because of this, the two chromophores are not strictly identical and SB-CS is no longer only controlled by solvent fluctuations.

All these effects play advantageously in the “sweet spot” geometry to achieve quantitative CS with a relatively long lifetime.

The structure/property relationship found here with **Pe** dimers, can be expected to be largely valid for the other dyes that are often used as building blocks in multichromophoric architectures. Therefore, these principles and quantitative correlations could be used advantageously for optimising the CS properties of such systems.

## Conflicts of interest

There are no conflicts to declare.

## Supplementary Material

SC-010-C9SC03913A-s001
