# Supplementary material for: Tuning symmetry breaking charge separation in perylene bichromophores by conformational control
Source: Chem Sci. 2019 Sep 30;10(45):10629–39. doi: 10.1039/c9sc03913a (PMC8133027; doi:10.1039/c9sc03913a)
Supplement: SC-010-C9SC03913A-s001 [file SC-010-C9SC03913A-s001.pdf]

Electronic Supplementary Material (ESI) for Chemical Science.
This journal is © The Royal Society of Chemistry 2019

Tuning symmetry breaking charge separation in perylene bichromophores by conformational control.

Alexander Aster,[a] Giuseppe Licari,[b] Francesco Zinna,[c] Elodie Brun,[c] Tatu Kumpulainen,[a] Emad Tajkhorshid,[b] Jérôme Lacour,[c] and Eric Vauthey*[a]

Electronic Supplementary Information

Contents

1 Experimental Details 2
1.1 Chemicals . . . 2
1.2 Steady State Measurements . . . 2
1.2.1 Absorption . . . 2
1.2.2 Emission . . . 2
1.3 Transient Absorption (TA) . . . 2
1.3.1 General Remarks . . . 2
1.3.2 fs-ps Pump . . . 2
1.3.3 ps-μs Pump . . . 2
1.3.4 Visible Probe . . . 2
1.3.5 Near Infrared Probe . . . 2
1.3.6 Data Treatment . . . 3
1.4 Broadband Fluorescence Up-converison Spectroscopy (FLUPS) . . . 3

2 Molecular Dynamics Simulations 3

3 Additional schemes 5
3.1 Mixing of states and the effect of asymmetry . . . 5
3.2 Conformers . . . 5

4 Additional Results 6
4.1 Host-Guest Complexes . . . 6
4.2 Stationary Spectroscopy . . . 8
4.2.1 Ref in DCM . . . 8
4.2.2 18c6 in ACN . . . 8
4.2.3 18c6 in DCM . . . 9
4.3 Molecular Dynamics Simulations . . . 10
4.3.1 18c6 in ACN . . . 10
4.3.2 18c6 in DCM . . . 10
4.3.3 18c4 in DCM . . . 11
4.3.4 16c4 in DCM . . . 12
4.3.5 Ref in DCM . . . 12
4.4 Transient Absorption . . . 13
4.4.1 Salt concentration . . . 13
4.4.2 18c6 in ACN . . . 13
4.4.3 18c6 in toluene . . . 14
4.4.4 18c6 in DCM . . . 15
4.4.5 Ref in DCM . . . 16
4.5 Global lifetime analysis of transient absorption data . . . 17
4.6 Broadband Fluorescence Up-conversion Spectroscopy . . . 19
4.6.1 18c6 in ACN . . . 19
4.6.2 18c6 in ACN and toluene . . . 19

1 Experimental Details

1.1 Chemicals

Acetonitrile (ACN, Roth, ≥ 99.9%), dichloromethane (DCM, Roth, ≥ 99.%), toluene (TOL, Acros, ≥ 99.8%) were used as received. Sodium tetrakis[3,5-bis(trifluoromethyl)phenyl]borate ($NaBAr_F$, Sigma-Aldrich) and $Ba(ClO_4)_2$ (Sigma-Aldrich, 97%) were used as received and stored in a desiccator.

1.2 Steady State Measurements

1.2.1 Absorption

Absorption spectra were measured on a Cary 50 spectrometer.

1.2.2 Emission

Emission spectra were measured on a Horiba Scientific FluoroMax-4 fluorometer and corrected using a set of secondary emissive standards.[1]

1.3 Transient Absorption (TA)

1.3.1 General Remarks

The TA data presented in this work were recorded with three different experimental setups: a fs-ps visible (fs-VIS), a fs-ps near infrared (fs-NIR) and a ps-μs visible (ps-VIS) TA setup. A detailed description of the general principle of the fs-ps as well as ps-μs TA applying referenced detection using two spectrographs is presented elsewhere.[2] In the following, experimental details concerning the three setups and the data analysis are discussed. The fs-VIS and fs-NIR setups share the same pump path, whereas the design of the fs-VIS and the ps-VIS probe path are identical. The absorbance of the sample at the excitation wavelength was 0.2-0.4 (at 400 nm) on 1 mm. The absorption spectra of all samples before and after the transient absorption experiments showed no signs of degradation. The samples in DCM were measured in a 1 mm quartz cuvettes (Starna, model 1GS/Q/1) and bubbled with nitrogen during the measurements giving a wavelength dependent IRF of about 80-350 fs (fwhm of optical Kerr effect (OKE)). The samples in ACN were measured with a flow cell with 200 μm, C7980 5F windows and a spacer thickness of 400 μm giving a wavelength dependent IRF of about 80-120 fs (fwhm of OKE).

1.3.2 fs-ps Pump

Excitation is performed using 400 nm pulses generated by frequency doubling part of the output of a 1 kHz Ti:Sapphire amplified system (Spectra Physics, Solstice Ace). The TA signal was checked prior to the experiment to scale linearly with the pump energy. Samples were excited with ca. 0.2-0.5 mJ cm^{-2} pump intensity. The polarization of the pump pulses was set to magic angle relative to the white-light pulses. In order to check for pump beam divergence and/or delay-line misalignment, the fs-TA dynamics of a calibration sample (perylene in DMSO) were compared to the dynamics measured on a time-correlated single photon counting setup.

1.3.3 ps-μs Pump

The ps-μs pumping was described in detail in ref. 3. Excitation was performed at 355 nm using a passively Q-switched, frequency doubled Nd:YAG laser (Teem Photonics, Powerchip NanoUV) producing pulses with a 500 Hz repetition rate, approximately 20 μJ energy per pulse, and 300 ps duration.

1.3.4 Visible Probe

Probing was achieved using white light pulses generated by focusing the 800 nm pulses of the Ti:Sapphire amplified system in a CaF_2 plate. The experimental setup was the same as that described earlier,[4] except that all lenses, after white light generation, were replaced by spherical mirrors to prevent chromatic aberration.

1.3.5 Near Infrared Probe

The white light was generated by focusing the 800 nm pulses in a YAG crystal. To balance the intensity of the white light, the high intensity light at 800 nm was removed by a beam stop after generation as well as by a 1 mm cuvette containing IR140 in DMSO. The probe light was then separated into a reference and a sample beam by a reflective metallic neutral density filter. After passing the sample, the beam was dispersed by a home-built prism spectrometer and the intensity recorded with a InGaAs detector. To balance the white light spectrum, apodizing neutral density filters were placed directly before both detectors.

1.3.6 Data Treatment

The pixel to wavelength conversion was achieved using a standard containing rare earth metals (NIST 2065 for NIR and holmium oxide for VIS) which shows narrow bands from the UV to the NIR. All transient absorption spectra were corrected for background signals showing up before time zero (e. g. spontaneous emission).
The fs-ps spectra were corrected for the dispersion due to the optical chirp using the optical Kerr effect.[5]
For merging the fs-vis and the fs-nir spectra, the TA signal in the overlap region between 690 and 740 nm was compared and one of the two datasets was multiplied with a constant factor accounting for the difference in pump power. The comparison of the kinetics recorded in the overlap region additionally serves as quality control, directly revealing erroneous kinetics e.g. due to deviation from the magic angle or poor alignment of the delay stage.

1.4 Broadband Fluorescence Up-converison Spectroscopy (FLUPS)

Broadband FLUPS measurements were performed on a setup similar to that described in detail in refs. 6, 7. In brief, excitation was performed with a 100 fs pulses at 400 nm generated by frequency doubling part of the output of a standard 1 kHz Ti:Sapphire amplified system. The pump intensity on the sample was below 1 mJ/cm^2. The gate pulses at 1340 nm were produced by an optical parametric amplifier (TOPAS Prime, Light Conversion). Detection of the up-converted spectra was performed with a home-built spectrograph coupled to a CCD camera (Andor, DV420A-BU). The full width at half-maximum (FWHM) of the cross correlation of the gate with the solvent Raman signal was approximately 170 fs. Time-resolved emission spectra were recorded in two sequential measurements with a linear time grid from –2 to 2 ps and with a logarithmic time grid extending up to 1.5 ns. The measurement consisted of 10 successive scans with 2 s collection time at each time step.The raw data were transferred into spectra vs. wavenumber and subsequently corrected by calibration with secondary emissive standards as described in ref. 6. The chirp due to group velocity dispersion was determined by measuring the instantaneous response of BBOT in the same solvent.

2 Molecular Dynamics Simulations

Classical molecular dynamics (MD) simulations were performed using NAMD2[8] in combination with the CHARMM36 force field.[9,10] Topology files for all chromophores and ions were retrieved from the CHARMM General Force Field (CGenFF),[11] and those for solvents (acetonitrile, ACN, and dichloromethane, DCM) were obtained through the *virtualchemistry.org* website.[12] Since the default DCM parameters generated a solvent environment with a very low dielectric constant (ϵ_r = 2.7), partial charges of DCM were further refined to more closely reproduce the experimental ϵ_r value of 8.9. For this optimisation, the restricted electrostatic potential (RESP) method[13] was used, providing the Merz-Singh-Kollman electrostatic potential[14] calculated with Gaussian 09 (Rev.D)[15] at the MP2/6-31+G(d) level of theory in the gas phase. After this refinement, the dipole moment of DCM raised to 2.1 D and the dielectric constant reached a value of 7.8. The optimised DCM partial charges are $\delta_C = -0.312$, $\delta_H = +0.226$ and $\delta_{Cl} = -0.070$.

For all production simulations, non-bonded interactions were evaluated with a cutoff of 12 Å, with a switching function starting at 10 Å. Long-range electrostatic interactions were accounted for by the particle mesh Ewald (PME)[16] method with a grid density of 1 Å^{-3} and a PME interpolation order of 6. All bonds involving hydrogen atoms were kept rigid using the SHAKE algorithm.[17] Simulations were performed in an NPT ensemble employing Nosé-Hoover thermostat and Langevin piston (1 atm, period: 50 fs, decay: 25 fs, damping coefficient 1.0 ps^{-1}).[18,19] MD time-step was set to 2 fs and both non-bonded and PME forces were updated at every time-step. Trajectories were visualized and analysed by VMD.[20] The data were collected every 4 ps and were analysed by in-house scripts. In particular, four intramolecular parameters were extracted from the trajectories (Figure S1): α: the angle between the two **Pe** long axes, i.e., the angle between the transition dipole moment of each individual **Pe** unit; β: the angle between the normals to the **Pe** planes; γ: the slip angle, defined as the angle between the long axis of one **Pe** moiety and the axis connecting the geometric centres of the two **Pe** moieties; and δ: the distance between the geometric centres of the two **Pe** groups.
Each simulated system contained one of the bichromophores of interest (**18c6**, **18c4** or **16c4**) immersed in a solvent box (ACN or DCM) with a side of 50 Å. We also prepared a control simulation in which two monomers (**Ref**) were placed in the same simulation box in order to reproduce the same number of **Pe** moieties as in the covalently-linked chromophores. The estimated concentration of the monomers in the box was 26 mM, which is higher than the experimental concentration used in this work (about 0.1 mM). Each MD system was replicated 5 times and each replica simulated in production mode for 21 ns. The first nanosecond of each replica was discarded. The remaining 20 ns of each replica per system were combined (overall 100 ns) and used for subsequent analysis. For the simulation containing barium cation, a sulfate ion was used to neutralize the overall charge of the system. In order to prevent a strong interaction between the sulfate and the chromophore due to the finite volume of the solvent box, the position of the sulfate was kept fixed in space and the partial charges of sulfur and oxygen in the sulfate were reduced to 0.288 and -0.572, respectively (initial values were 0.992 and -0.748). In all other simulations containing cations, we placed two Na^+ so to have the same amount of positive ions in solution as for the simulations

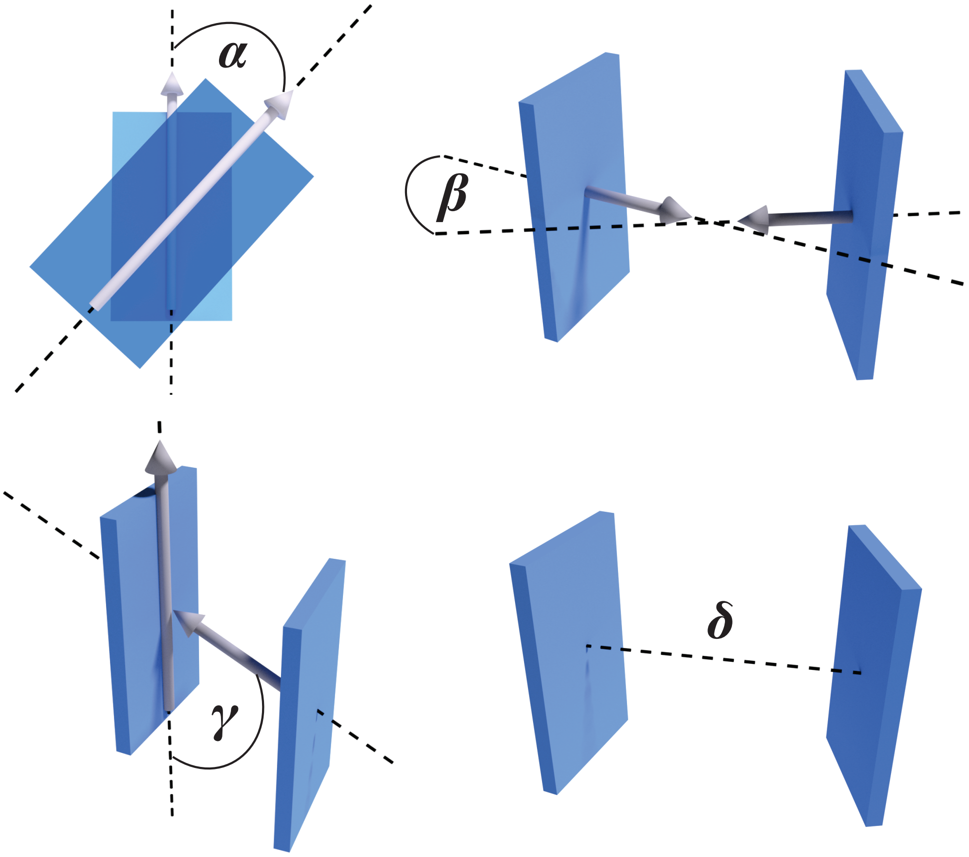

Figure S1: Intramolecular parameters extracted from MD trajectories (blue rectangles represent **Pe** moieties): α) the angle between the two **Pe** long axes, β) the angle between the normals to the **Pe** planes, γ) the angle between the long axis of one **Pe** moiety and the axis connecting the geometric centres of the two **Pe** heads, and δ) the distance between the geometric centres of the two **Pe** groups.

containing barium. The two positive sodium ions were neutralized with a sulfate as for barium and the positions of sulfate and the sodium ion not involved in the interaction with the crown ether were fixed in space. Since the formation of the host:guest (HG) complex by equilibrium MD is computationally expensive, initial structures for such complexes were prepared by placing the cation at the geometric centre of each crown ether, minimizing for 500 steps and running short simulations with positional restraints of decreasing force constants on the complex at each simulation. This procedure provided HG complexes where the cation remained inside the crown-ether cavity stably during the simulations without applying any restraint.

For the simulations involving the 1:2 complex of **18C6** and Na^+ in DCM, both sodium cations and the sulfate were free to move. However, two NBFIX[21] parameter sets were added between 1) sulfate oxygens and sodium cations, and 2) sulfate oxygens and amide hydrogens, in order to avoid short-range interactions of the sulfate with the complex. Parameters for both NBFIX were ϵ = -0.1 kcal/mol and R_{min}= 20 Å. In the starting configuration for the 1:2 complex, both sodium cations were placed inside the crown ether on the same plane as its oxygen atoms. After few hundreds of picoseconds of equilibration, a single Na^+ occupied the crown ether cavity and the second sodium cation interacted with the amide groups adopting the conformation described in the main text.

3 Additional schemes

3.1 Mixing of states and the effect of asymmetry

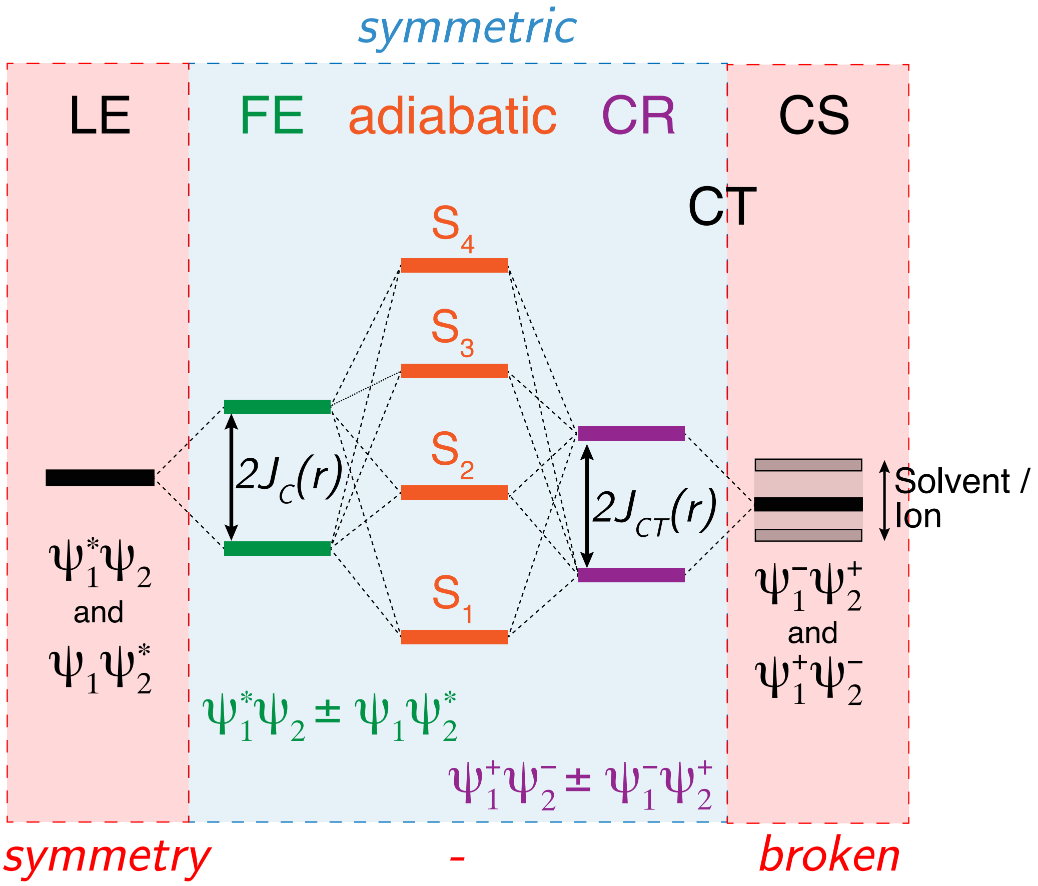

Figure S2: Mixing towards the adiabatic states starting from the diabatic basis of the monomers in vacuum at a fixed interchromophore conformation. The local environment (solvent or salt) can lift the degeneracy of the charge separated states and therefore lead to symmetry breaking as indicated in grey. LE: locally excited; FE: Frenkel exciton; CR: charge resonance; CT: charge transfer; CS: charge separation.

3.2 Conformers

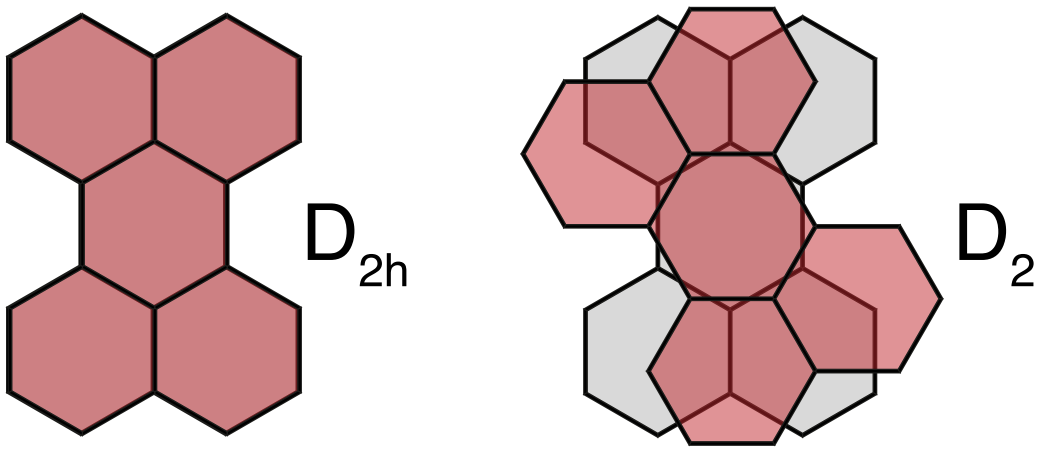

Figure S3: Perylene dimer conformations. The eclipsed (D_{2h}) conformation is calculated to be the excited state minimum (excimer) whereas the 30° rotated (D_2) conformer is the ground state minimum.[22,23]

4 Additional Results

4.1 Host-Guest Complexes

In order to keep the host concentration constant a stock solution (ST1) of the sample (bichromophore or **Ref**) was prepared in the respective solvent . ST1 was then used to dissolve a certain amount of salt giving the stock solution (ST2). ST2 was then titrated to ST1 and the spectral changes were recorded after each addition.
Due to a limited availability of the samples, the molar absorption coefficient and therefore the concentration of the host could not be accurately determined. To estimate the host concentration, a molar absorption coefficient of 34500 $M^{-1}cm^{-1}$, which is the literature value[24] for the parent **Pe**, was used for the maximum of **Ref** upon complexation of the cation. A molar absorption coefficient of 69000 $M^{-1}cm^{-1}$ was assumed for the low coupling bichromophores, which showed the signatures of a local excited state in the transient absorption spectra. The association constants as well as the spectra obtained from the global analysis of the absorption spectra with varying guest (salt) concentration are depicted in Table S1 and Figure S4, respectively. It has to be noted that for very high association constants as observed for the bichromophores, the error on the host concentration has a strong influence on the association constant. Therefore, the reported constants should only be used to compare the complexation behaviour between the different crown-ethers and **Ref**.

Table S1: Association constants determined from global analysis.

Species	Solvent	Salt	Model	K1 / M^{-1}	K2 / M^{-1}
**18c6**	ACN	$Ba(ClO_4)_2$	1:1	$4 \cdot 10^7$	-
**18c6**	DCM	$NaBAr_F$	1:2	$2 \cdot 10^6$	$1 \cdot 10^3$
**18c4**	DCM	$NaBAr_F$	1:1	$2 \cdot 10^6$	-
**16c4**	DCM	$NaBAr_F$	1:1	$1 \cdot 10^6$	-
**Ref**	DCM	$NaBAr_F$	1:1	$1 \cdot 10^4$	-

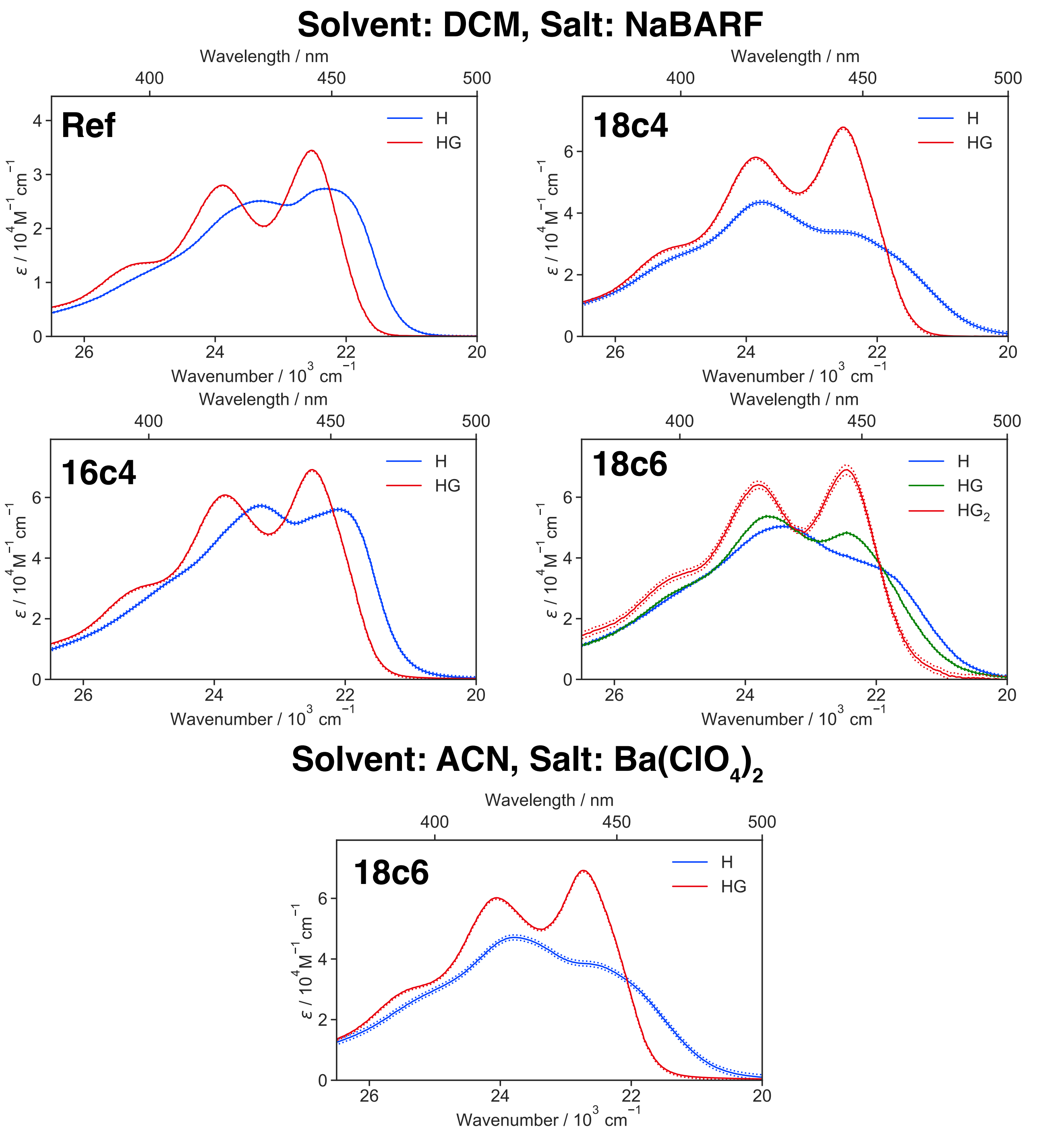

Figure S4: Molar absorption coefficients of the bichromophores in DCM and ACN obtained from a global analysis of the absorption spectra at different guest (cation) concentrations. The dotted lines indicate the 95 % confidence interval obtained from the global analysis.

4.2 Stationary Spectroscopy

4.2.1 Ref in DCM

The stationary absorption and emission spectra, before and after addition of $NaBAr_F$ are shown in Figure S5. Details on the complexation are discussed in section 4.1.

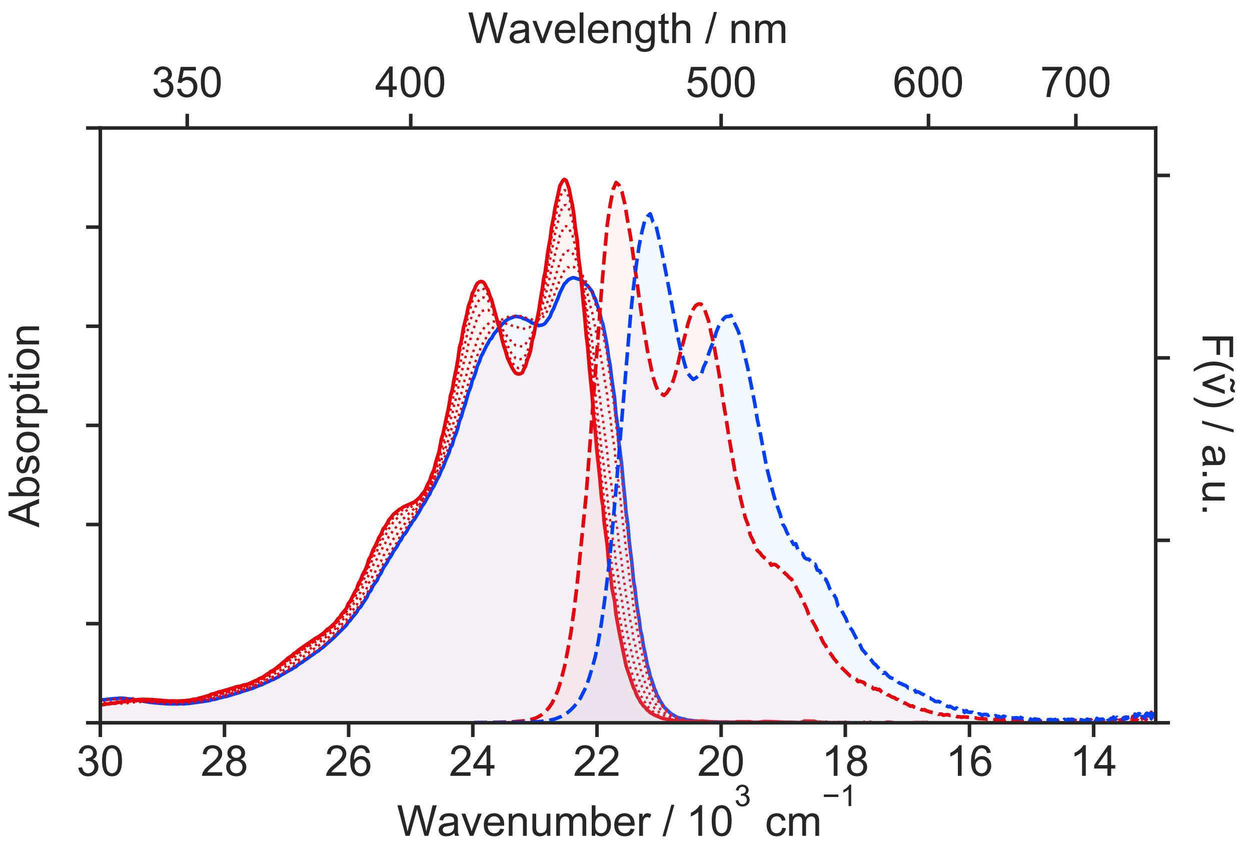

Figure S5: Absorption and emission spectra of **Ref** in DCM with (red) and without (blue) binding of Na^+.

4.2.2 18c6 in ACN

The excitation/emission map of **18c6** in ACN before (A), and after complexation of Ba_2^+ is shown in Figure S6. Slices of this dataset are illustrated in Figure S7 depicting the wavelength dependence of excitation/emission of the emission/excitation spectra.

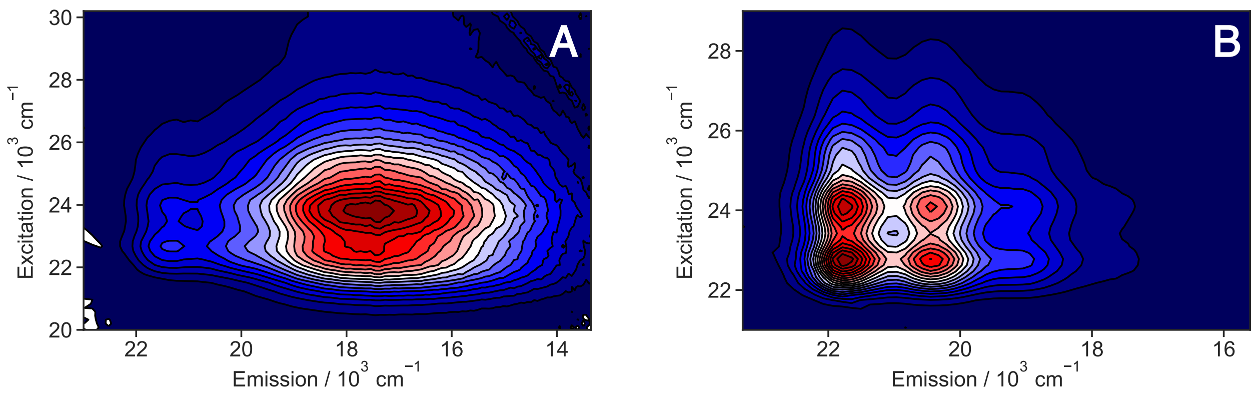

Figure S6: Excitation/emission map of **18c6** in ACN (A) and spectral changes upon addition of $Ba(ClO_4)_2$ (B).

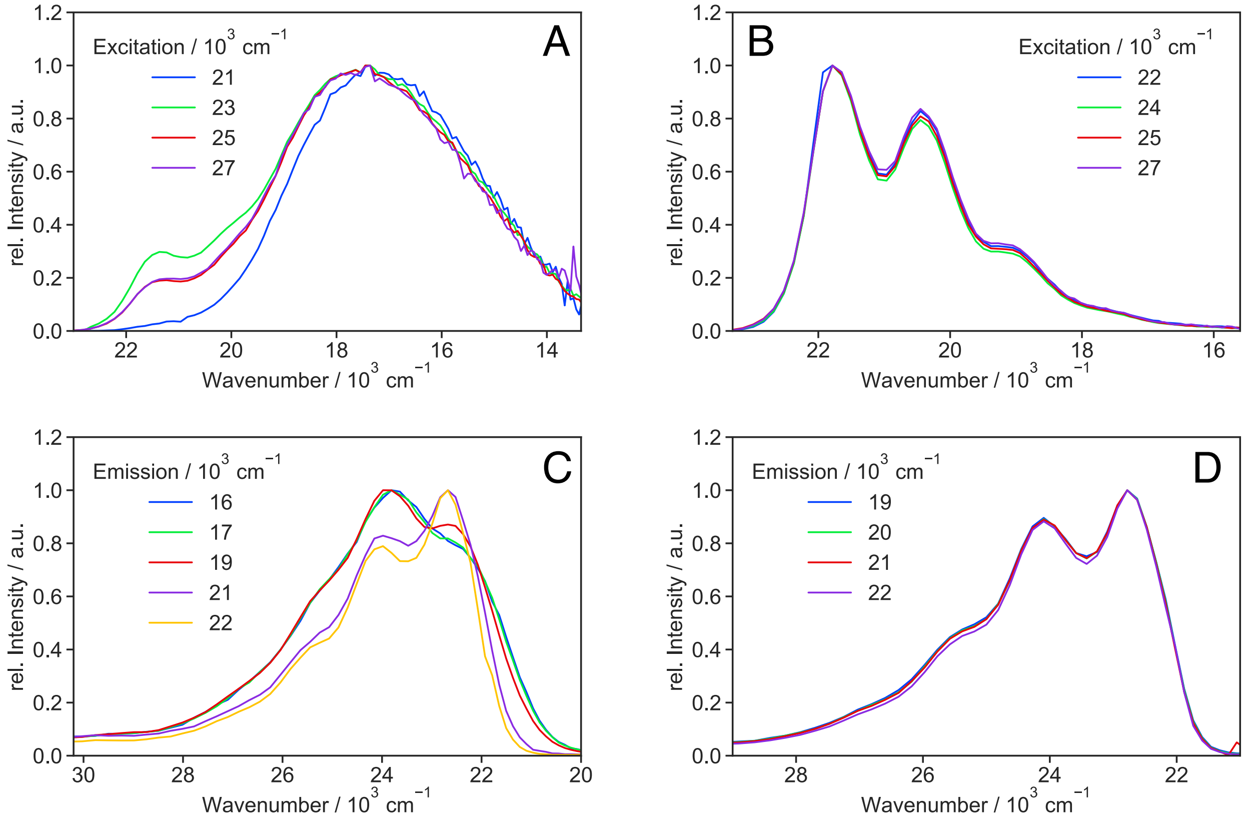

Figure S7: Emission (A) and excitation (C) spectra of **18c6** in ACN and spectral changes upon addition of $Ba(ClO_4)_2$ (B & D).

4.2.3 18c6 in DCM

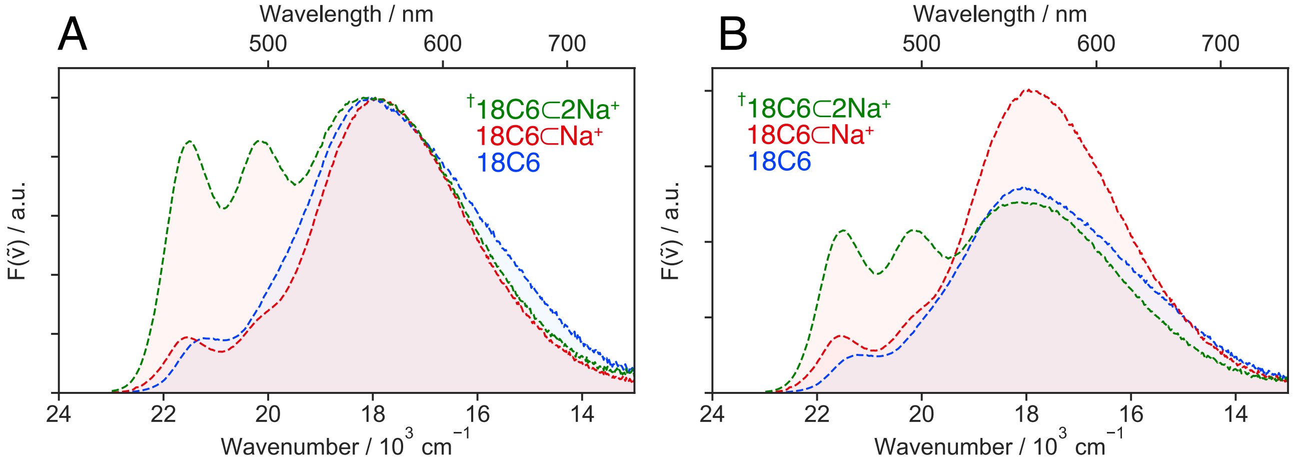

Figure S8: (A) Normalized emission spectra of **18c6** (blue), **18C6⊂Na**$^+$ (red) and †**18C6⊂2 Na**$^+$ (green). The red shifted, featureless bands of †**18C6⊂2 Na**$^+$ and **18C6⊂Na**$^+$ resemble each other and are therefore attributed to the **18C6⊂Na**$^+$ sub-population present in †**18C6⊂2 Na**$^+$. Furthermore, the excimer spectrum of **18c6** contains an even further red shifted part compared to the **18C6⊂Na**$^+$ spectrum. This could be due to weaker geometrical restrictions in **18C6** enabling the approach to a stronger coupled conformer and therefore a lower energy excimer. The absolute intensity (B) illustrates that the intensity of the excimer band increases upon formation of **18C6⊂Na**$^+$. This observation along with the absence of the NIR band in transient absorption suggest that the excimer conformations for **18C6** and **18C6⊂Na**$^+$ are substantially different whereas the coupling is stronger in the former and closer to the eclipsed D_{2h}.

4.3 Molecular Dynamics Simulations

4.3.1 18c6 in ACN

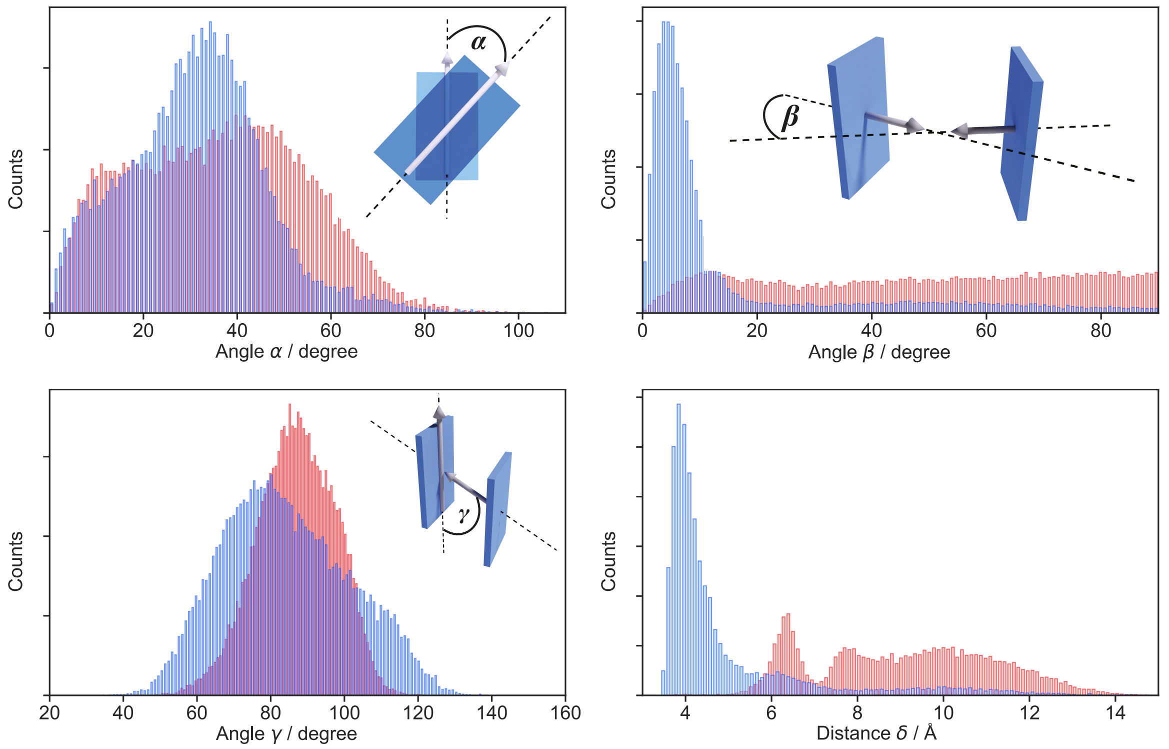

Figure S9: Histograms of different structural coordinates of **18c6** in ACN. **18c6** (blue); **18C6⊂Ba^{2+}** (red).

4.3.2 18c6 in DCM

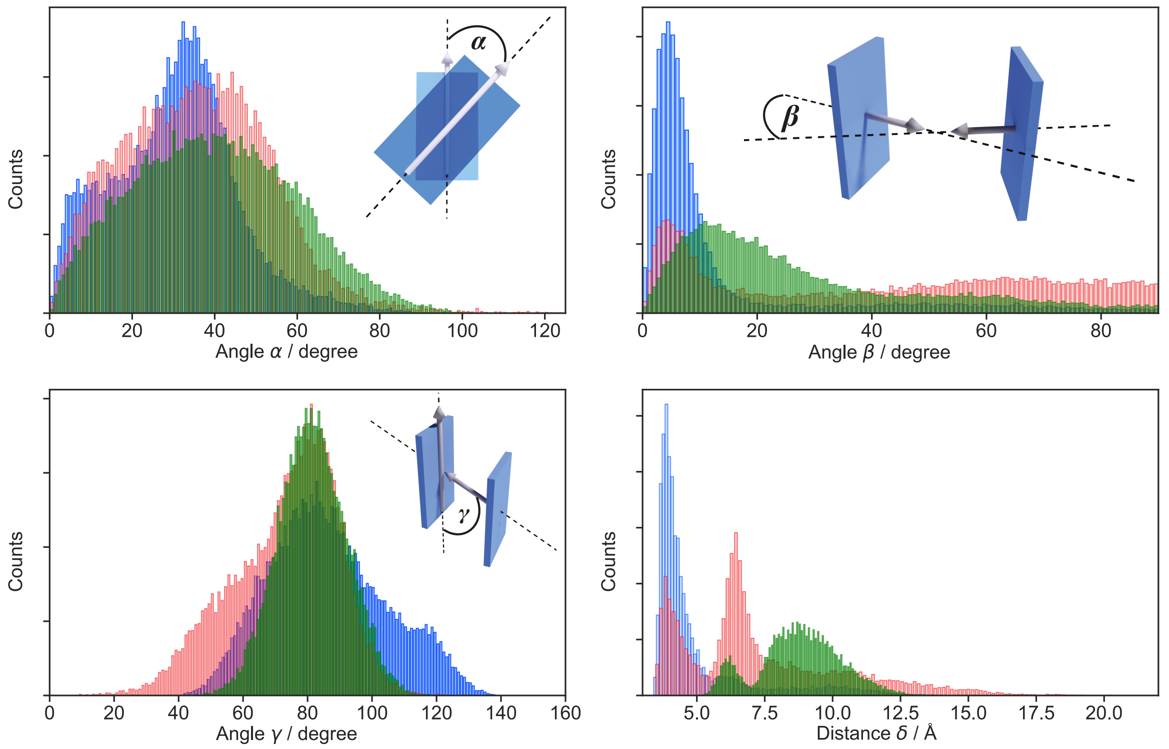

Figure S10: Histograms of different structural coordinates for **18c6** in DCM. **18c6** (blue); **18C6⊂Na^{+}** (red); **18C6⊂2 Na^{+}**(green).

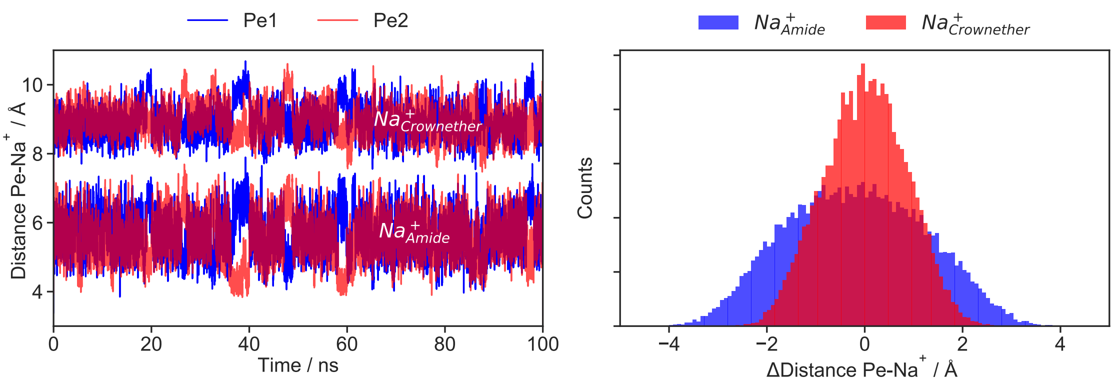

Figure S11: MD trajectories of the distances between the Na^+ cations (that bound to the crown-ether and that bound to the amide oxygens) and the centres of the **Pe** heads (left), and histograms of the difference between these distances (right).

4.3.3 18c4 in DCM

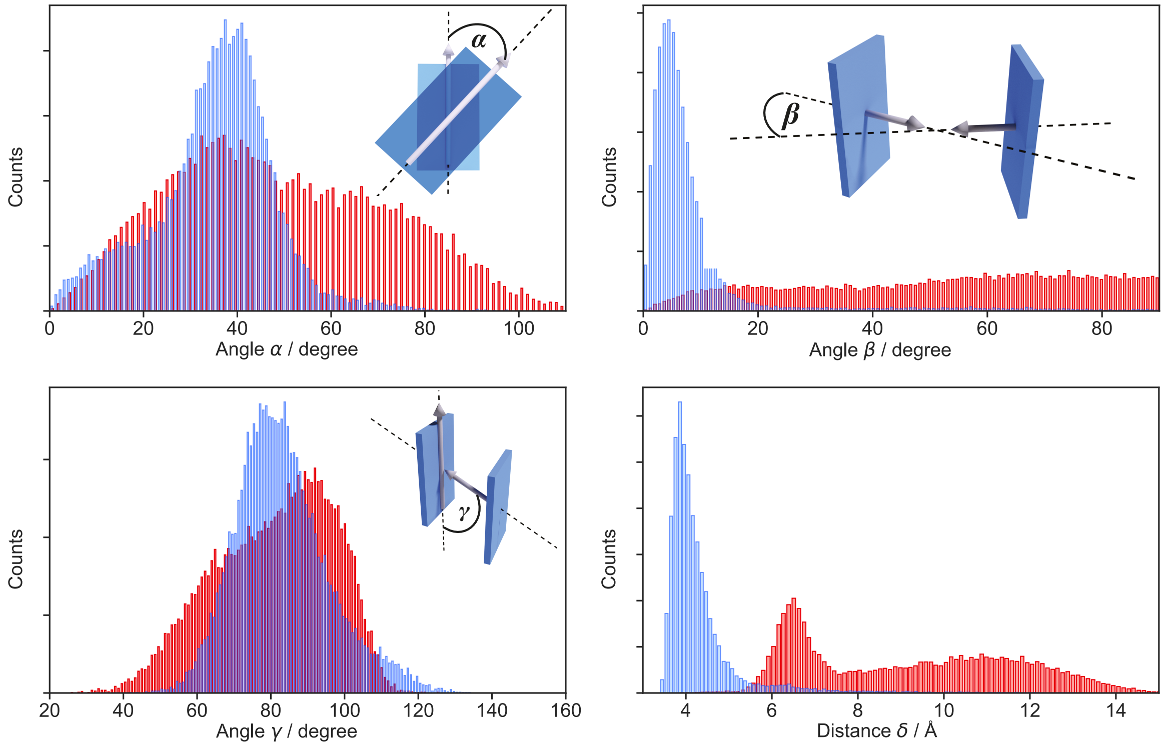

Figure S12: Histograms of different structural coordinates for **18c4** in DCM. **18c4** (blue); **18C4⊂Na**$^+$ (red).

4.3.4 16c4 in DCM

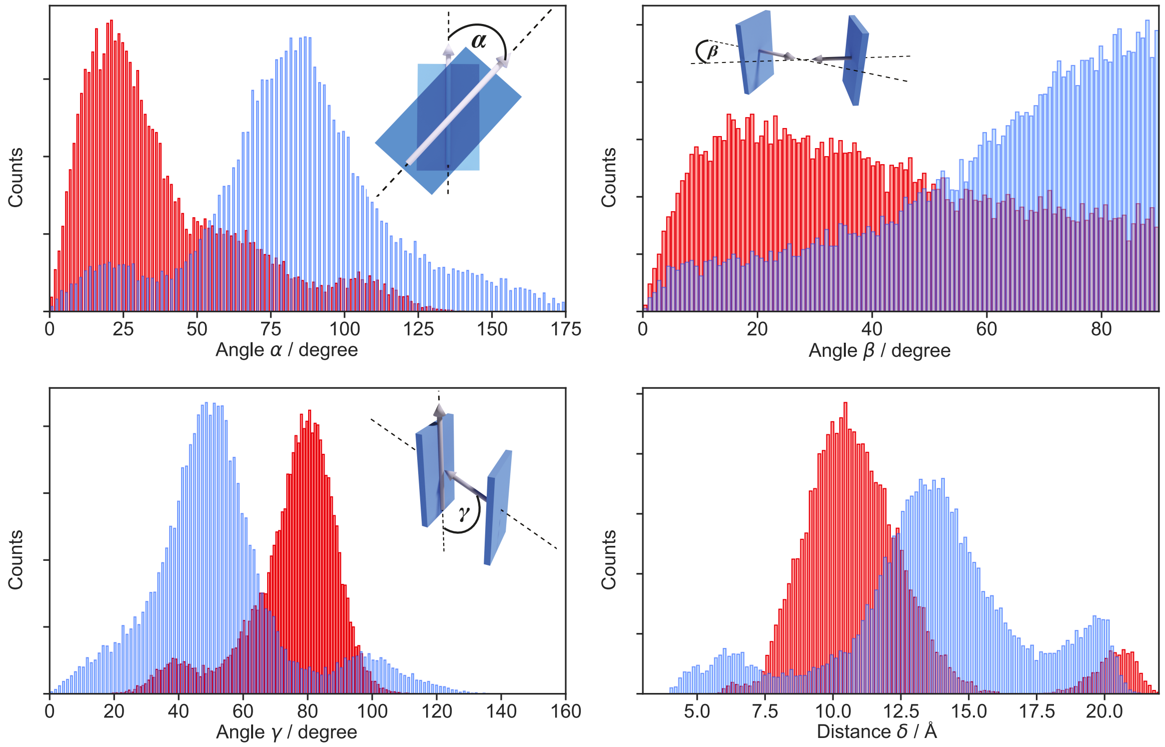

Figure S13: Histograms of different structural coordinates for **16c4** in DCM. **16c4** (blue); **16C4⊂Na$^+$** (red).

4.3.5 Ref in DCM

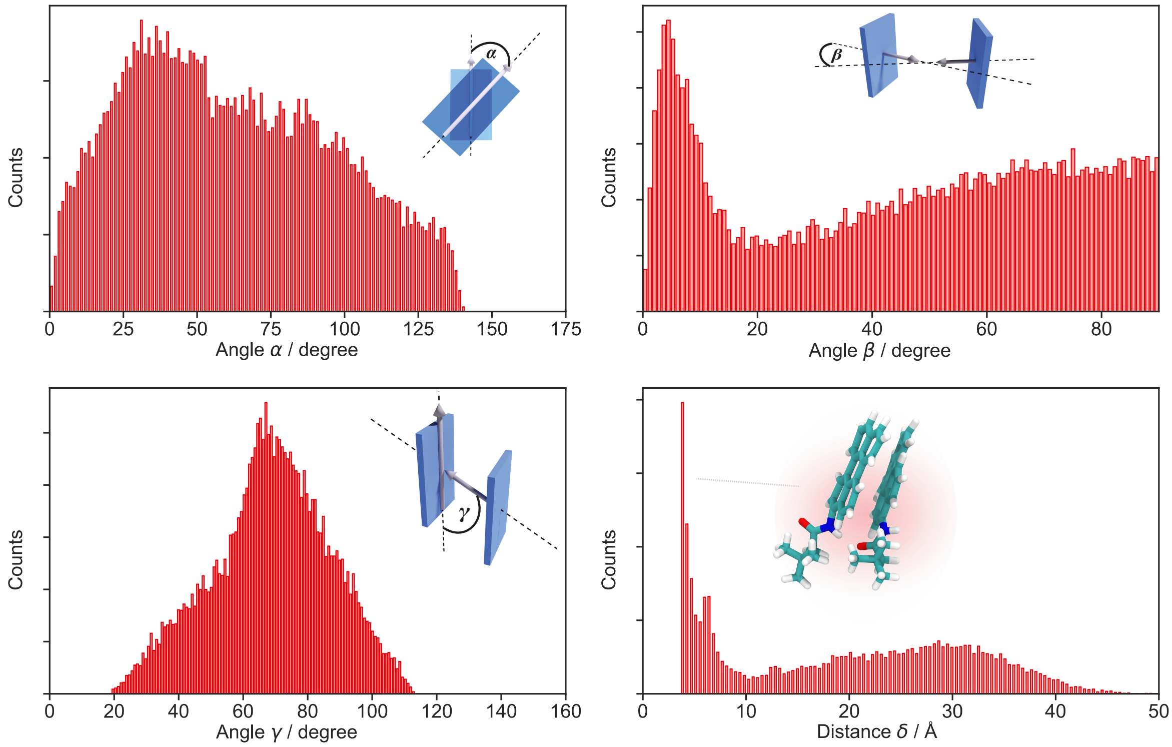

Figure S14: Histograms of different structural coordinates for two **Ref** molecules in DCM.

4.4 Transient Absorption

4.4.1 Salt concentration

The concentration of salt was adjusted to obtain at least 95 % 1:1 complexes, corresponding to about 100 μM. To obtain †**18C6⊂2 Na**$^{+}$, the concentration of $NaBAr_F$ was about 1.5 mM.

4.4.2 18c6 in ACN

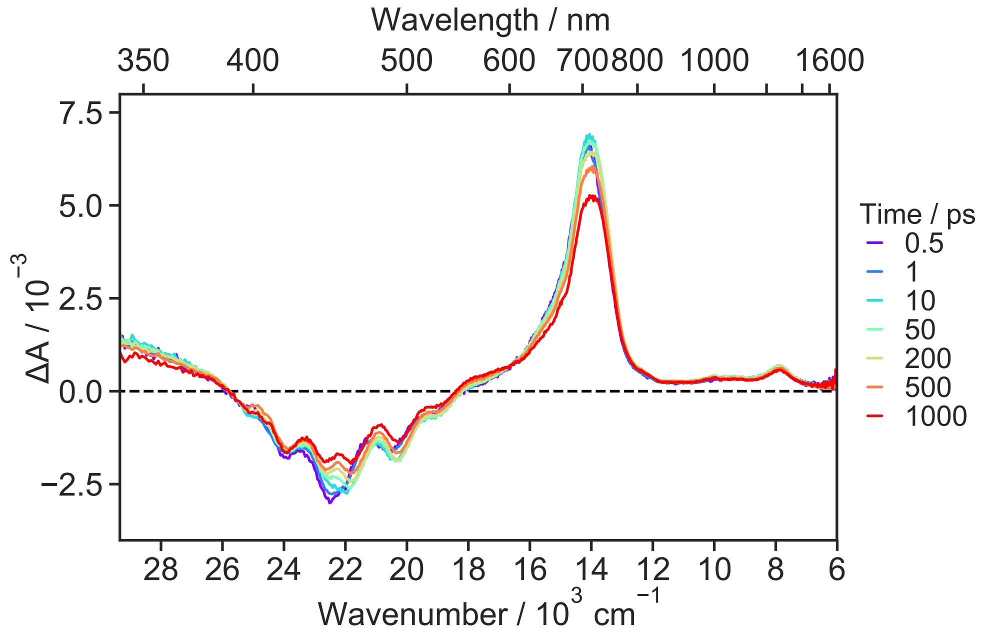

Figure S15: Transient absorption spectra in the UV/Vis/NIR regions measured at various time delays after excitation of **18C6⊂Ba**$^{2+}$ in ACN.

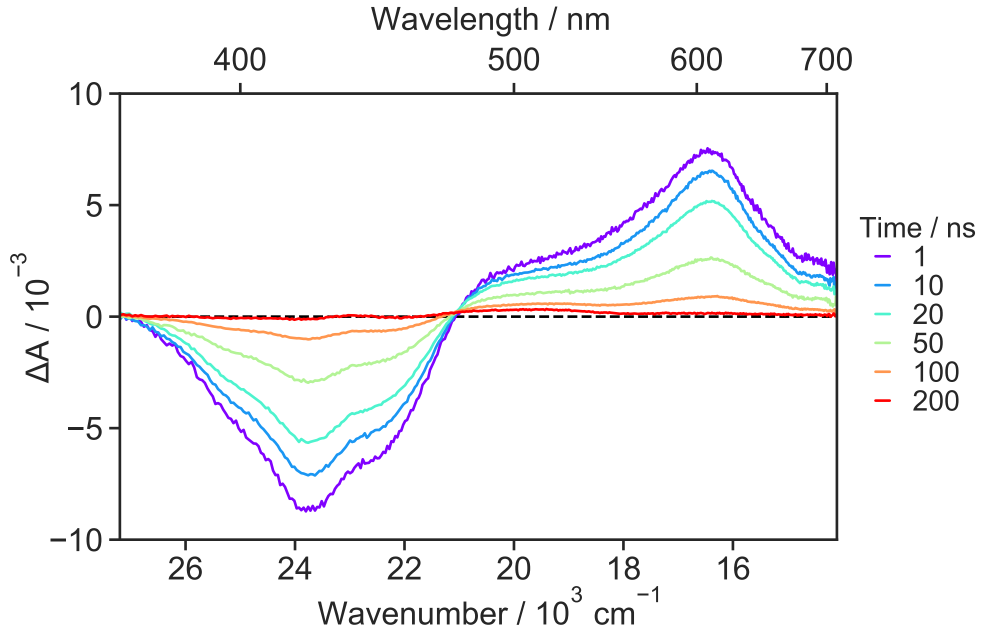

Figure S16: Transient absorption spectra in the UV/Vis regions measured at various time delays after excitation of **18c6** in ACN.

4.4.3 18c6 in toluene

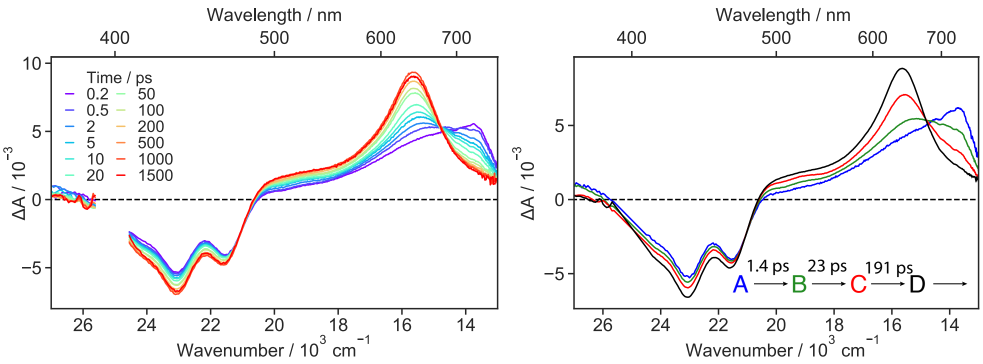

Figure S17: Transient absorption spectra in the UV/Vis regions measured at various time delays after excitation of **18c6** in toluene (left). Evolution associated difference spectra (EADS) with the corresponding time constants (right).

4.4.4 18c6 in DCM

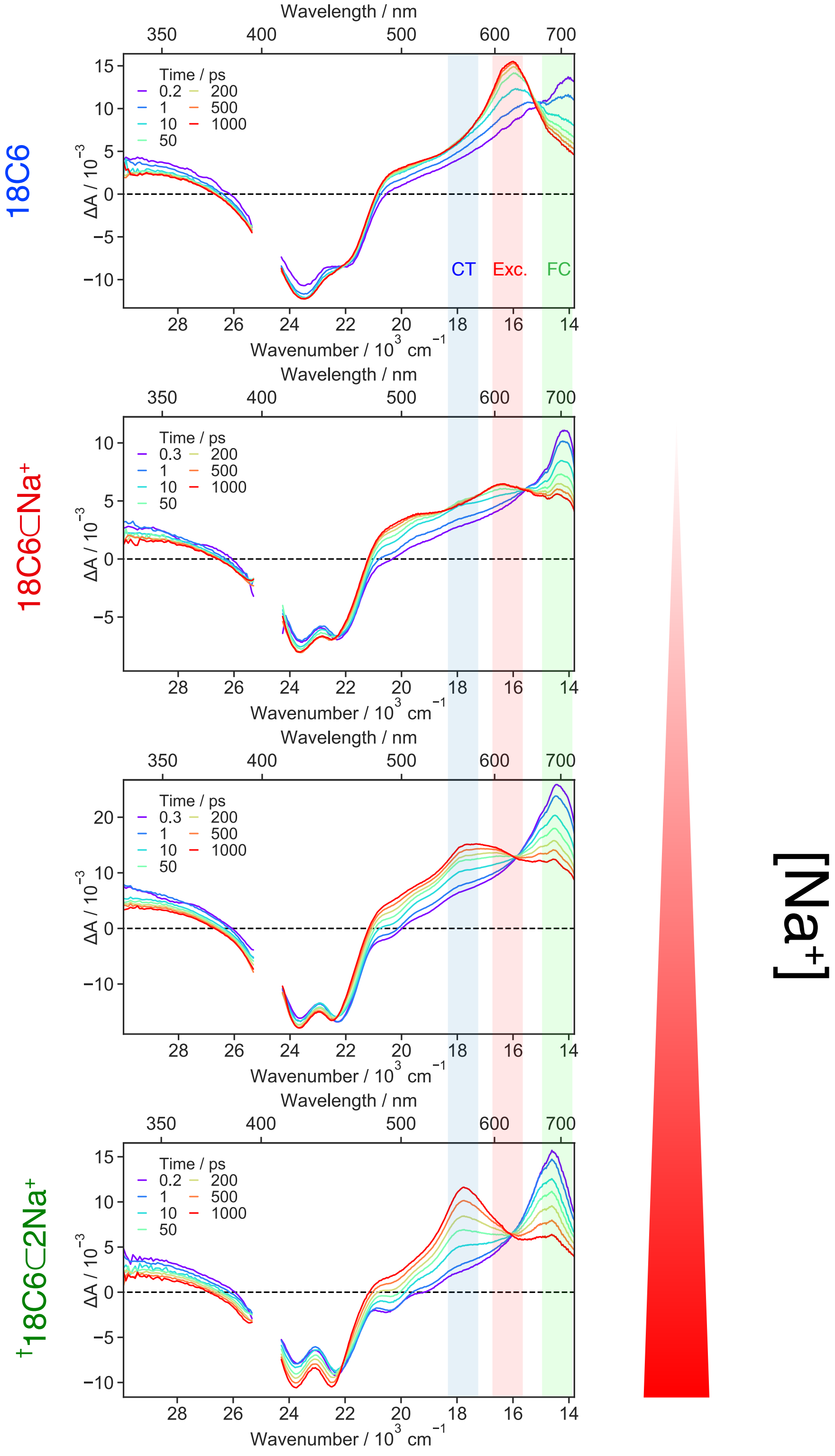

Figure S18: Transient absorption spectra in the UV/Vis region measured at various time delays after excitation of **18c6** in DCM at different Na^+ concentrations. Upon further addition of salt to **18C6⊂Na**$^+$, the **18C6⊂2 Na**$^+$/**18C6⊂Na**$^+$ ratio increases leading to an increase of the I_{0-0}/I_{1-0} ratio of the ground state bleach, an increase of the stimulated emission as well as the charge-transfer (CT) feature.

4.4.5 Ref in DCM

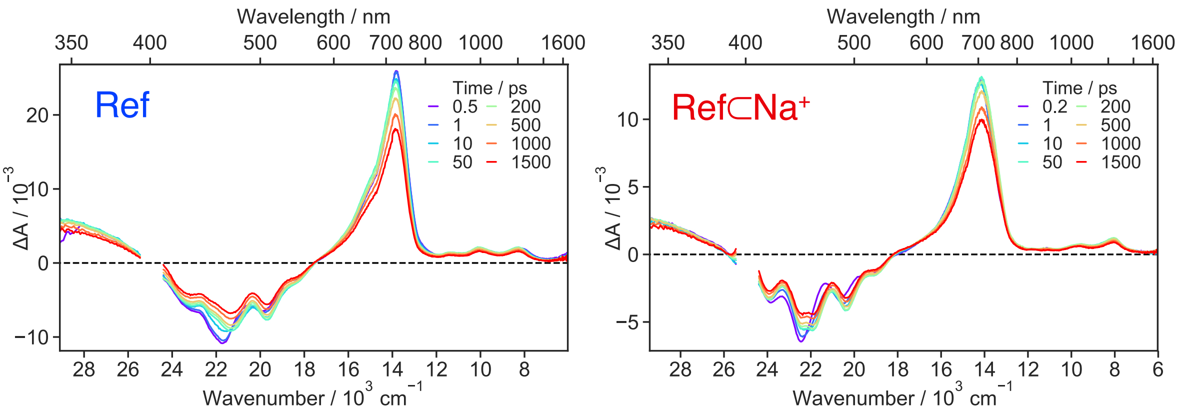

Figure S19: fs-ps transient absorption spectra in the UV/Vis/NIR regions measured after excitation of **Ref** in DCM.

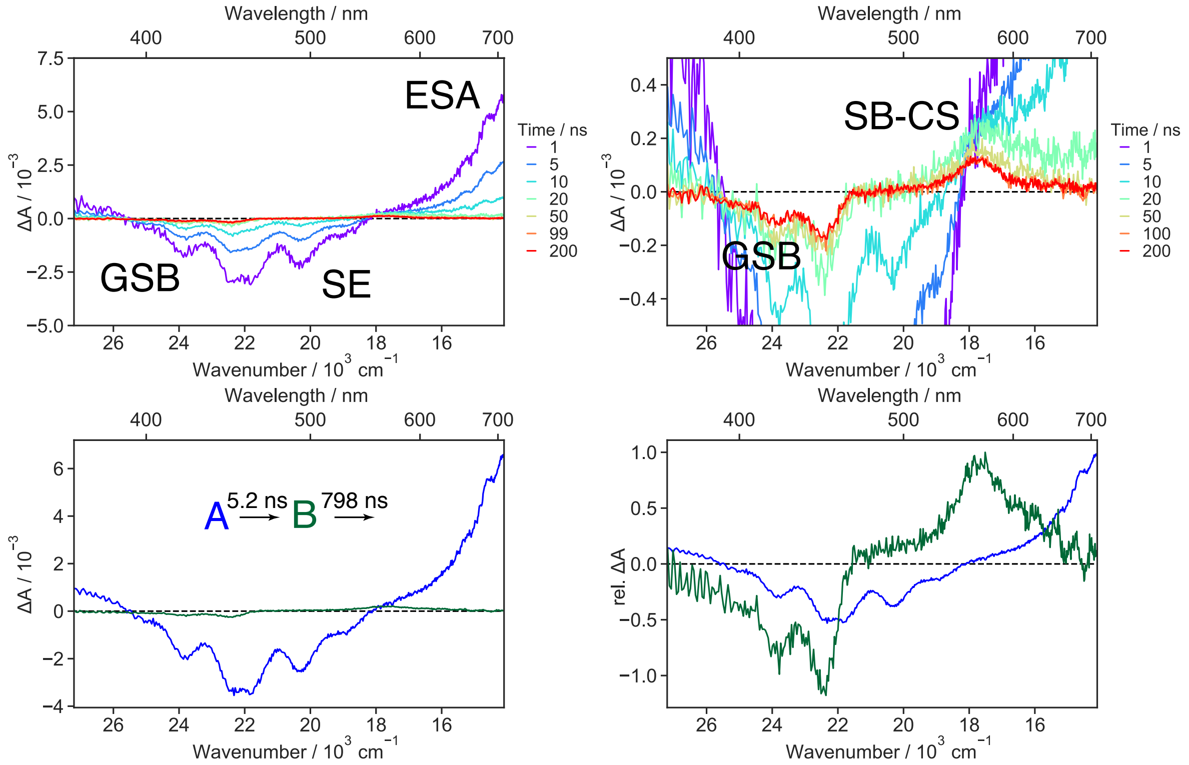

Figure S20: fs-μs transient absorption spectra illustrating the self-quenching of **Ref** in DCM by symmetry breaking charge separation.

4.5 Global lifetime analysis of transient absorption data

The broadband transient absorption data were analysed globally using a global lifetime analysis based on the approach discussed in detail in ref. 25, 26. We assumed a sequential model for all the datasets analysed. This corresponds to a single population that evolves as a series of n successive exponential steps into further species without any losses or back-reactions as illustrated in Figure S21. In this case, one can obtain the so-called Evolution Associated Difference Spectra (EADS), which represent the spectral evolution. However, the EADS do not systematically correspond to given species or states but only help to visualizing the timescales on which the spectral dynamics occur. The lifetimes and EADS are shown in Table S2 and Figure S22 .

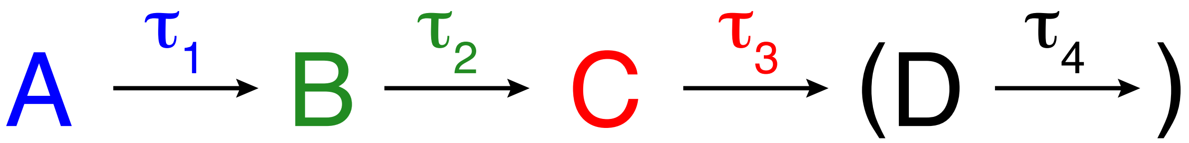

Figure S21: Sequential model used in the global analysis. The time constants are listed in Table S2 and the evolution associated spectra A-D are illustrated in Figure S22.

Table S2: Time constants obtained from the global analysis of the transient absorption data applying the successive model illustrated in Figure S21 .

Compound	Solvent	Bound Ion(s)	τ_1 / ps	τ_2 / ps	τ_3 / ps	τ_4 / ps
**18C6**	ACN		0.6	8.7	292	long
**18C6⊂Ba^{2+}**	ACN	Ba^{2+}	2.9	201	long	-
**18C6**	DCM		1.5	21	523	long
**18C6⊂Na^{+}**	DCM	Na^{+}	1.9	21	235	long
†**18C6⊂2 Na^{+}**	DCM	$2Na^{+}$	2.5	22	639	long
**18C4**	DCM		1.7	14	329	long
**18C4⊂Na^{+}**	DCM	Na^{+}	1.3	27	1600	long
**16C4**	DCM		3.4	82	852	long
**16C4⊂Na^{+}**	DCM	Na^{+}	1.3	31	1100	long
**Ref**	DCM		5.8	83	long	-
**Ref⊂Na^{+}**	DCM	Na^{+}	2.0	29	long	-

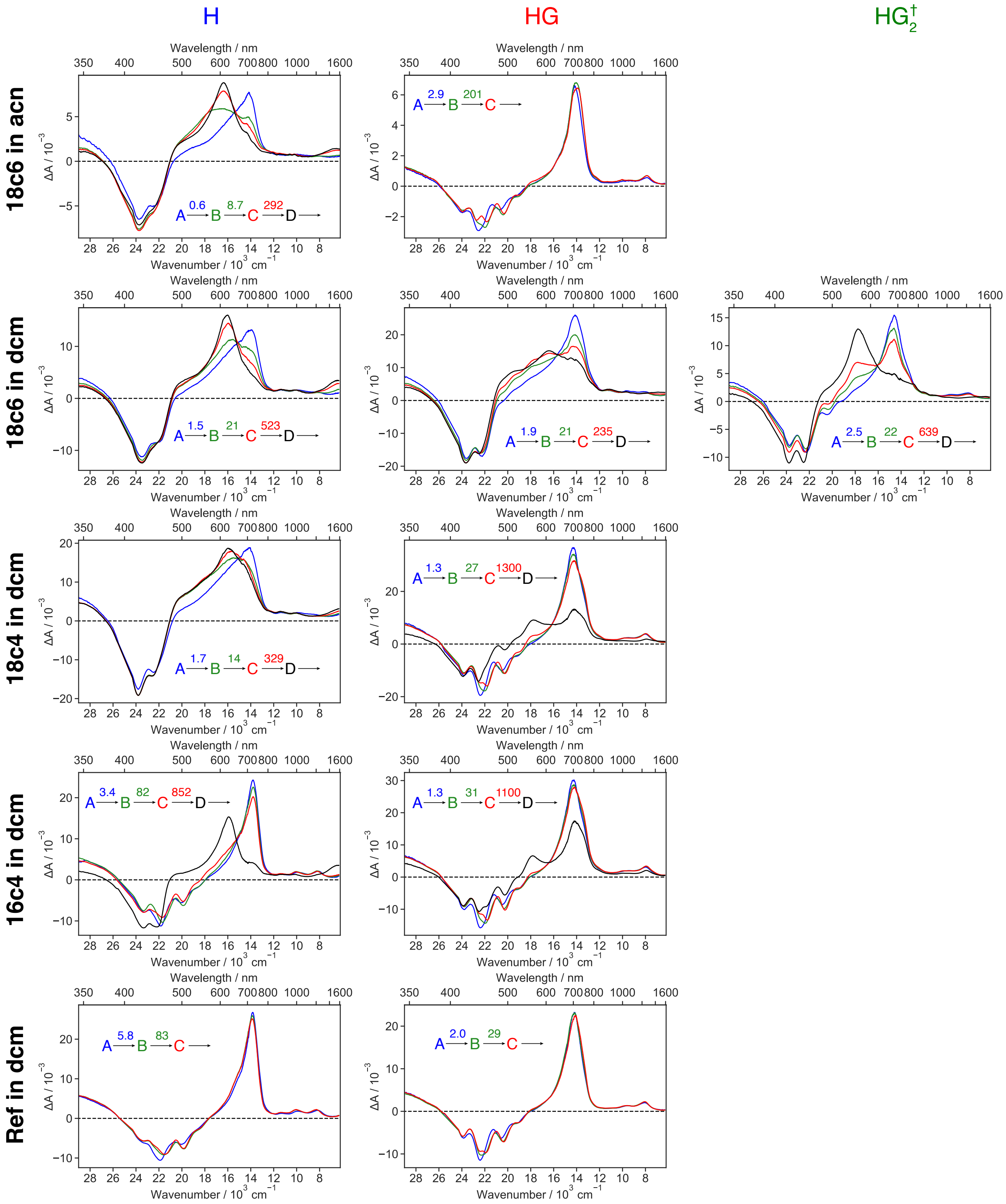

Figure S22: Evolution associated decay spectra (EADS) obtained from a global analysis of the transient absorption data.

4.6 Broadband Fluorescence Up-conversion Spectroscopy

4.6.1 18c6 in ACN

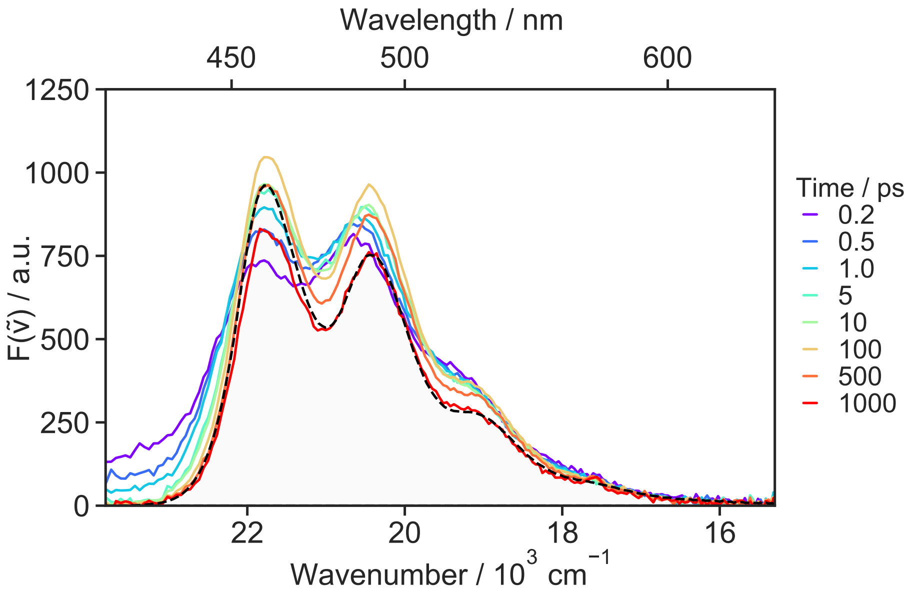

Figure S23: Fluorescence spectra recorded at various time delays after 400 nm excitation of **18c6** in the presence of Ba^{2+} in acetonitrile and stationary emission spectrum (dotted line). The difference between the stationary and the transient spectra at long time delay is due to the inner filter effect, which decreases the intensity of the 0-0 band.

4.6.2 18c6 in ACN and toluene

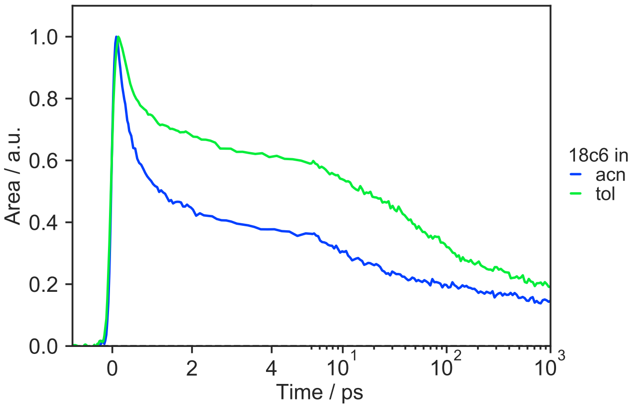

Figure S24: Time profiles of the fluorescence band area measured with **18c6** in ACN and toluene. Due to vibrational relaxation occurring during the first few ps, the area between 15500 and 23000 cm^{-1} was used. The faster decay in ACN is attributed to SB-CT that accelerates the decrease of the transition dipole moment.

References

[1] J. A. Gardecki and M. Maroncelli. Set of Secondary Emission Standards for Calibration of the Spectral Responsivity in Emission Spectroscopy. *Appl. Spectrosc.*, 52:1179–1189, 1998.

[2] B. Lang. Photometrics of Ultrafast and Fast Broadband Electronic Transient Absorption Spectroscopy: State of the Art. *Rev. Sci. Instrum.*, 89:093112, 2018.

[3] B. Lang, S. Mosquera-Vázquez, D. Lovy, P. Sherin, V. Markovic, and E. Vauthey. Broadband Ultraviolet-Visible Transient Absorption Spectroscopy in the Nanosecond to Microsecond Time Domain with Sub-Nanosecond Time Resolution. *Rev. Sci. Instrum.*, 84:073107, 2013.

[4] N. Banerji, G. Duvanel, A. Perez-Velasco, S. Maity, N. Sakai, S. Matile, and E. Vauthey. Excited-State Dynamics of Hybrid Multichromophoric Systems: Toward an Excitation Wavelength Control of the Charge Separation Pathways. *J. Phys. Chem. A*, 113:8202–8212, 2009.

[5] E. Tokunaga, A. Terasaki, and T. Kobayashi. Femtosecond continuum interferometer for transient phase and transmission spectroscopy. *J. Opt. Soc. Am. B*, 13:496–513, 1996.

[6] X.-X. Zhang, C. Würth, L. Zhao, U. Resch-Genger, N. P. Ernsting, and M. Sajadi. Femtosecond Broadband Fluorescence Upconversion Spectroscopy: Improved Setup and Photometric Correction. *Rev. Sci. Instrum.*, 82:063108, 2011.

[7] M. Gerecke, G. Bierhance, M. Gutmann, N. P. Ernsting, and A. Rosspeintner. Femtosecond Broadband Fluorescence Upconversion Spectroscopy: Spectral Coverage versus Efficiency. *Rev. Sci. Instrum.*, 87:053115, 2016.

[8] J. C. Phillips, R. Braun, W. Wang, J. Gumbart, E. Tajkhorshid, E. Villa, C. Chipot, R. D. Skeel, L. Kale, and K. Schulten. Scalable Molecular Dynamics with NAMD. *J. Comput. Chem.*, 26:1781–802, 2005.

[9] K. Hart, N. Foloppe, C. M. Baker, E. J. Denning, L. Nilsson, and Jr. Mackerell, A. D. Optimization of the CHARMM Additive Force Field for DNA: Improved Treatment of the BI/BII Conformational Equilibrium. *J. Chem. Theory Comput.*, 8:348–362, 2012.

[10] J. B. Klauda, R. M. Venable, J. A. Freites, J. W. O'Connor, D. J. Tobias, C. Mondragon-Ramirez, I. Vorobyov, Jr. MacKerell, A. D., and R. W. Pastor. Update of the CHARMM All-Atom Additive Force Field for Lipids: Validation on Six Lipid Types. *J. Phys. Chem. B*, 114:7830–43, 2010.

[11] K. Vanommeslaeghe, E. Hatcher, C. Acharya, S. Kundu, S. Zhong, J. Shim, E. Darian, O. Guvench, P. Lopes, I. Vorobyov, and Jr. Mackerell, A. D. CHARMM General Force Field: A Force Field for Drug-like Molecules Compatible with the CHARMM All-Atom Additive Biological Force Fields. *J. Comput. Chem.*, 31:671–90, 2010.

[12] C. Caleman, P. J. van Maaren, M. Hong, J. S. Hub, L. T. Costa, and D. van der Spoel. Force Field Benchmark of Organic Liquids: Density, Enthalpy of Vaporization, Heat Capacities, Surface Tension, Isothermal Compressibility, Volumetric Expansion Coefficient, and Dielectric Constant. *J. Chem. Theory Comput.*, 8:61–74, 2012.

[13] C. I. Bayly, P. Cieplak, W. Cornell, and P. A. Kollman. A Well-Behaved Electrostatic Potential Based Method Using Charge Restraints for Deriving Atomic Charges: The RESP Model. *J. Phys. Chem.*, 97:10269–10280, 1993.

[14] U. C. Singh and P. A. Kollman. An Approach to Computing Electrostatic Charges for Molecules. *J. Comput. Chem.*, 5:129–145, 1984.

[15] Michael J. Frisch, G. W. Trucks, H. Bernhard Schlegel, Gustavo E. Scuseria, Michael A. Robb, James R. Cheeseman, Giovanni Scalmani, Vincenzo Barone, Benedetta Mennucci, G. A. Petersson, H. Nakatsuji, M. Caricato, Xiaosong Li, H. P. Hratchian, Artur F. Izmaylov, Julien Bloino, G. Zheng, J. L. Sonnenberg, M. Hada, M. Ehara, K. Toyota, R. Fukuda, J. Hasegawa, M. Ishida, T. Nakajima, Y. Honda, O. Kitao, H. Nakai, T. Vreven, J. A. Montgomery Jr., J. E. Peralta, François Ogliaro, Michael J. Bearpark, Jochen Heyd, E. N. Brothers, K. N. Kudin, V. N. Staroverov, Rika Kobayashi, J. Normand, Krishnan Raghavachari, Alistair P. Rendell, J. C. Burant, S. S. Iyengar, Jacopo Tomasi, M. Cossi, N. Rega, N. J. Millam, M. Klene, J. E. Knox, J. B. Cross, V. Bakken, C. Adamo, J. Jaramillo, R. Gomperts, R. E. Stratmann, O. Yazyev, A. J. Austin, R. Cammi, C. Pomelli, J. W. Ochterski, R. L. Martin, K. Morokuma, V. G. Zakrzewski, G. A. Voth, P. Salvador, J. J. Dannenberg, S. Dapprich, A. D. Daniels, Ödön Farkas, J. B. Foresman, J. V. Ortiz, J. Cioslowski, and Douglas J. Fox. Gaussian 09 (Rev. C and D). 2009.

[16] T. Darden, D. York, and L. Pedersen. Particle Mesh Ewald: An N·log(N) Method for Ewald Sums in Large Systems. *J. Chem. Phys.*, 98:10089, 1993.

[17] J.P. Ryckaert, G. Ciccotti, and H. J. C. Berendsen. Numerical Integration of the Cartesian Equations of Motion of a System with Constraints: Molecular Dynamics of n-Alkanes. *J. Comput. Phys.*, 23:327–341, 1977.

[18] G. J. Martyna, D. J. Tobias, and M. L. Klein. Constant Pressure Molecular Dynamics Algorithms. *J. Chem. Phys.*, 101:4177–4189, 1994.

[19] S. E. Feller, Y. Zhang, R. W. Pastor, and B. R. Brooks. Constant Pressure Molecular Dynamics Simulation: The Langevin Piston Method. *J. Chem. Phys.*, 103:4613–4621, 1995.

[20] W. Humphrey, A. Dalke, and K. Schulten. VMD: Visual Molecular Dynamics. *J. Mol. Graph.*, 14:33–38, 1996.

[21] R. M. Venable, Y. Luo, K. Gawrisch, B. Roux, and R. W. Pastor. Simulations of Anionic Lipid Membranes: Development of Interaction-Specific Ion Parameters and Validation using NMR Data. *J. Phys. Chem. B*, 117, 2013.

[22] D. Casanova. Theoretical Investigations of the Perylene Electronic Structure: Monomer, Dimers, and Excimers. *Int. J. Quant. Chem.*, 115:442–452, 2015.

[23] V. Settels, W. Liu, J. Pflaum, R. F. Fink, and B. Engels. Comparison of the Electronic Structure of Different Perylene-Based Dye-Aggregates. *J. Comput. Chem.*, 33:1544–1553, 2012.

[24] G. Angulo, A. Rosspeintner, B. Lang, and E. Vauthey. Optical Transient Absorption Experiments Reveal the Failure of Formal Kinetics in Diffusion Assisted Electron Transfer Reactions. *Phys. Chem. Chem.Phys.*, 20:25531–25546, 2018.

[25] I. H. M. van Stokkum, D. S. Larsen, and R. van Grondelle. Global and Target Analysis of Time-Resolved Spectra. *Biochim. Biophys. Acta, Bioenerg.*, 1657:82–104, 2004.

[26] P. Fita, E. Luzina, T. Dziembowska, Cz. Radzewicz, and A. Grabowska. Chemistry, Photophysics, and Ultrafast Kinetics of Two Structurally Related Schiff Bases Containing the Naphthalene or Quinoline Ring. *J. Chem. Phys.*, 125:184508, 2006.
